# Elevated Cytokines, Thrombin and PAI-1 in Severe HCPS Patients Due to Sin Nombre Virus

**DOI:** 10.3390/v7020559

**Published:** 2015-02-10

**Authors:** Virginie Bondu, Ron Schrader, Mary Ann Gawinowicz, Paul McGuire, Daniel A. Lawrence, Brian Hjelle, Tione Buranda

**Affiliations:** 1 Department of Pathology, University of New Mexico School of Medicine, Albuquerque, NM 87131, USA; E-Mails: vbondu@salud.unm.edu (V.B.); bhjelle@salud.unm.edu (B.H.); 2 Clinical and Translational Science Center, University of New Mexico Health Sciences Center, Albuquerque, NM 87131, USA; E-Mail: rschrader@salud.unm.edu; 3 Herbert Irving Comprehensive Cancer Center, Columbia University, New York, NY 10031, USA; E-Mail: mag4@columbia.edu; 4 Cell Biology and Physiology, University of New Mexico School of Medicine, Albuquerque, NM 87131, USA; E-Mail: pmcguire@salud.unm.edu; 5 Department of Internal Medicine, University of Michigan Medical School, Ann Arbor, MI 48109-5644, USA; E-Mail: dlawrenc@med.umich.edu

**Keywords:** hantavirus, PAI-1, ECIS, cell barrier function, hemostasis, proteomics

## Abstract

Sin Nombre Hantavirus (SNV, *Bunyaviridae Hantavirus*) is a Category A pathogen that causes Hantavirus Cardiopulmonary Syndrome (HCPS) with case fatality ratios generally ranging from 30% to 50%. HCPS is characterized by vascular leakage due to dysregulation of the endothelial barrier function. The loss of vascular integrity results in non-cardiogenic pulmonary edema, shock, multi-organ failure and death. Using Electric Cell-substrate Impedance Sensing (ECIS) measurements, we found that plasma samples drawn from University of New Mexico Hospital patients with serologically-confirmed HCPS, induce loss of cell-cell adhesion in confluent epithelial and endothelial cell monolayers grown in ECIS cultureware. We show that the loss of cell-cell adhesion is sensitive to both thrombin and plasmin inhibitors in mild cases, and to thrombin only inhibition in severe cases, suggesting an increasing prothrombotic state with disease severity. A proteomic profile (2D gel electrophoresis and mass spectrometry) of HCPS plasma samples in our cohort revealed robust antifibrinolytic activity among terminal case patients. The prothrombotic activity is highlighted by acute ≥30 to >100 fold increases in active plasminogen activator inhibitor (PAI-1) which, preceded death of the subjects within 48 h. Taken together, this suggests that PAI-1 might be a response to the severe pathology as it is expected to reduce plasmin activity and possibly thrombin activity in the terminal patients.

## 1. Introduction

Hantaviruses cause hemorrhagic fever with renal syndrome (HFRS) and hantavirus cardiopulmonary syndrome (HCPS, also called HPS) which can collectively cause over 100,000 cases per annum worldwide [1,2,3,4,5]. All of the etiologic agents associated with cardiopulmonary syndrome are found in the Western Hemisphere with the most important etiologic agents include Sin Nombre Hantavirus (SNV, *Bunyaviridae Hantavirus*) in the United States and Canada and Andes virus in Chile and Argentina [6,7,8]. Case fatality ratios for HCPS generally range from 30% to 50%. No vaccines or specific therapy are currently available for use in the US.

HCPS is characterized by vascular leakage due to dysregulation of endothelial barrier function [3,9]. After entry of SNV through inhalation, infected individuals experience an asymptomatic incubation period of one to six weeks, followed by a period of prodromal illness including headache, nausea, vomiting, myalgias and fever. This is then followed by profound dyspnea and shortness of breath due to noncardiogenic pulmonary edema (capillary leak) [3]. Ensuing death generally results from low-output cardiogenic shock, and rarely from hypoxia [10,11]. There is no approved, effective therapy and treatment of severe disease is supportive, including the use of extracorporeal membrane oxygenation (ECMO), a capability that is typically available to no more than a few adult patients at a time at some of the larger tertiary care centers [12]. The mechanism by which hantaviruses cause vascular permeability is poorly understood and appears to involve several factors [13,14,15].

This study used Electric Cell-substrate Impedance Sensing (ECIS) measurements to examine the effects, on cell-cell adhesion, of plasma samples drawn from UNM Hospital patients with HCPS. We found that plasma samples from patients with acute HCPS induce loss of cell barrier function in confluent epithelial and endothelial cell monolayers grown in ECIS cultureware. We show that the magnitude of loss of cell-cell adhesion depends on disease severity and is sensitive to thrombin and plasmin inhibitors. A proteomic profile (2D gel electrophoresis and mass spectrometry) of plasma samples from patients in the acute stages of HCPS revealed significant up and down regulation of proteolytic products of the complement, coagulation and fibrinolysis systems highlighted by a broad-range change of ≥30 to >100-fold increase in active plasminogen activator inhibitor (PAI-1) in terminal case-patients. We suggest that the extremely high levels of PAI-1 might be a response to the severe pathology, where PAI-1 is expected to limit plasminogen activation and possibly thrombin activity in terminal-case patients [16,17,18].

## 2. Experimental Section

### 2.1. Study Subjects

Coded plasma samples from 14 HCPS patients admitted to the University of New Mexico hospital between 2005 and 2007 were used in this retrospective study. Samples were previously collected with written informed consent, approved by the Human institutional review board of the University of New Mexico HSC. Patients with serologically proven SNV infections ranged from those having mild-moderate disease (no cardiopulmonary failure and did not require mechanical ventilation) to severe disease (pulmonary failure and hemodynamic compromise requiring mechanical ventilation and/or extracorporeal membrane oxygenation (ECMO) due to cardiac insufficiency). For this study, patients were stratified into three categories according to the severity of illness, with Class I (*n* = 5) exhibiting mild symptoms, whereas Class II (*n* = 5) and III (*n* = 4) patients were typically subjected to ECMO; with Class II representing those who survived while Class III represented fatal cases [19]. The plasma samples, were originally separated from venous blood collected in EDTA tubes, and stored in aliquots at −80 °C within an hour of collection. This study examined admission day samples for class I, and serial samples collected for up to 5 days from admission in class II and III. Control samples were derived from a pool of 10 healthy individuals.

### 2.2. Materials

Rabbit polyclonal H319 anti DAF antibody was purchased from Santa Cruz Biotechnology. Secondary antibodies: (anti-mouse IgG Alexa488, anti-mouse IgG Alexa647, anti-mouse IgG Cy5, anti-rabbit Alexa647; all from Molecular Probes). Phosphate-buffered saline (PBS) was purchased from Mediatech, Inc., (Herndon, VA, USA). Dimethyl sulfoxide (DMSO) and Sephadex G-50 were purchased from Sigma. TRIS (10 mM or 25 mM Tris, 150 mM NaCl, pH 7.5) and HHB (30 mM HEPES, 110 mM NaCl, 10 mM KCl, 1 mM MgCl_2_6H_2_O and 10 mM glucose, pH 7.4) buffer, and Hanks Balanced Saline Solution (HBSS) (0.35 g NaH_2_CO_3_, 0.049 g MgSO_4_, 1 mM CaCl_2_ or 1 mM MnCl_2_) were prepared under sterile conditions and stored in 50 mL tubes at −20 °C.

### 2.3. Cell Culture

Vero E6 were maintained in Dulbecco’s Modified Eagle Medium (DMEM) (GIBCO, Grand Island, NY, USA). All media contain 10% heat-inactivated fetal bovine serum (FBS), 100 units/mL penicillin, 100 μg/mL streptomycin, 10 mM HEPES, pH 7.4, 20 μg/mL ciprofloxacin, 2 mM l-glutamine, at 37 °C in a water jacketed 5% CO_2_ incubator. Human umbilical Vein Endothelial cells (HUVEC), Human Lung Microvascular Endothelial Cell (HLMVEC) and Telomerase-Immortalized human umbilical Vein Endothelial (TIVE) [20] were maintained in EBM-2 Basal Medium with EGM-2 SingleQuot Kit supplements and growth factors (Lonza, Walkersville, MD, USA).

### 2.4. Production of Sin Nombre Virus

SNV was propagated and titered in Vero E6 cells under strict standard operating procedures using biosafety level 3 (BSL3) facilities and practices (CDC registration number C20041018-0267) as previously described [21,22].

### 2.5. Measurement of Transmonolayer Cell Resistance Electric Cell-Substrate Impedance Sensing

Electric Cell-substrate Impedance Sensing (ECIS) is capable of detecting and quantifying morphology changes in the sub-nanometer to micrometer range [23,24]. For ECIS measurement, cells were seeded at 10^5^ cells/cm^2^ onto fibronectin-coated gold microelectrodes in ECIS cultureware (8W10E^+^) and incubated overnight at 37 °C (Zθ; Applied Biophysics, Troy, NY, USA). Vero E6 or endothelial cells: HUVEC, HLMVEC and TIVE cells were allowed to attach, spread, and organize for at least 24 h. Cellular impedance was measured continuously at a single frequency of 4000 Hz. Vero E6 epithelial cells derived from monkey kidney cells form more robust cell-cell barrier contacts (with typical resistance values in the 2000–4000 ohm range depending on passage) compared to endothelial cells (1000–1500 ohms), which are often difficult to culture in large quantities on a consistent basis. Vero cells are therefore less sensitive to spurious environmental cues that disrupt cell barrier function, and used in our application as a robust platform for ECIS measurements. When cellular impedance reached plateau values at 3000–4000 Ω for Vero 6 cells and 1000–1500 Ω for endothelial cells, patient and control plasma samples were added to each well. The data from duplicate or triplicate wells were averaged and presented as normalized resistance *versus* time.

### 2.6. Transmonolayer Electrical Resistance Measurements of Monolayer Integrity

Transmonolayer electrical resistance measurements (TER) were used to assess the barrier integrity of tight junctions in polarized cells. Vero and TIVE cells were plated at 150,000 cells per 6 mm transwell insert in 24 well plates (Corning, Tewksbury, MA, USA) in appropriate medium and allowed to develop tight junctions for 4 to 8 days. Formation of tight junctions was evaluated by measuring an increase in transmonolayer electrical resistance (TER) across the cell monolayer every 2 days using a voltohmeter coupled to an Endohm sensor chamber (World Precision Instruments Inc., Sarasota, FL, USA) at 37 °C. The measured potential difference between the upper and the lower chambers was used to calculate the electrical resistance in Ω cm^2^ (measured resistance *x* area of membrane), by subtracting the baseline electrical resistance measurement of polycarbonate filters in the absence of a cell monolayer.

### 2.7. TER and Infectivity Assays

TER measurements in a BSL3 lab were used to examine changes in monolayer integrity in response to activation with live SNV strain SN77734 inocula (moi = 0.1). To block binding of SNV to cells, the virus particles were mixed with excess soluble DAF for 1 h as previously described [25]. As an alternative, cells were treated with Reopro [26,27]. Virus inocula were then added to the apical chamber of polarized monolayer surfaces in a serum-free medium. TER measurements were carried out every 5 min for 30 min using a slide warmer to maintain 37 °C temperature of the transwell chambers.

### 2.8. Plasma Cytokines and Chemokines

Cytokines were measured in plasma samples using a HCYTMAG-60K-PX41 Cytokine kit following protocols supplied by the manufacturer (Millipore). Duplicate measurements were analyzed for significance using a two-way ANOVA with a Bonferroni posttest using GraphPad Prism software (San Diego, CA, USA). Overall our statistical approach is described in the context of 2D gel spot data below.

### 2.9. Confocal Microscopy Imaging

Tight junction markers occludin or ZO1 were imaged in cell monolayers with Zeiss META or LSM 510 systems using 63 × 1.4 oil immersion objectives as previously described [28]. One hundred fifty thousand cells were plated at confluency in 8-well Nunc Lab-Tek chambers (thermoscientific.com) or in inverted transwell filter supports and allowed to propagate for 2 days. At room temperature, cells were fixed with 3% paraformaldehyde for 20 min, and subsequently permeabilized with 0.2% Triton X100 (in PBS) for 15 min. Cells were blocked with 1% BSA in PBS for 30 min and then incubated with either anti ZO1 (rabbit polyclonal from Abcam, 1:100 dilution in blocking buffer) or occludin (mouse monoclonal antibody from Invitrogen, 1:200 dilution in blocking buffer) overnight at 4 °C. Cells were then incubated with a suitable Alexa488 tagged secondary antibody (Millipore, 1:100 dilution in blocking buffer for 1.5 h at room temperature). Samples were washed and imaged on the confocal microscope.

### 2.10. 2D Gel Loading and Sample Preparation

Samples were diluted in binding buffer from the Proteoextract Albumin/IgG removal Kit from CalbioChem (Cat #122642) and the Albumin and IgG removed. Samples were then concentrated using Amicon Ultra 10 k membrane centrifuge filters using 15 min centrifugations of 14,000 RPM at 4 °C. The protein concentrations of the samples were then determined using the BCA Assay [29]. Two-dimensional electrophoresis was performed by Kendrick Labs, Inc. (Madison, WI, USA) using the carrier ampholine method of isoelectric focusing [30]. Isoelectric focusing was carried out in a glass tube of inner diameter 3.3 mm using 2% pH 3–10 Isodalt Servalytes (Serva, Heidelberg, Germany) for 20,000 Vh. One hundred ng of an IEF internal standard, tropomyosin, was added to the sample. This protein migrates as a doublet with lower polypeptide spot of MW 33,000 and pI 5.2; an arrow on the stained gel marks its position (Figure S5 in supplementary materials). After equilibration for 10 min in Buffer “O” (10% glycerol, 50 mM dithiothreitol, 2.3% SDS and 0.0625 M tris, pH 6.8), each tube gel was sealed to the top of a stacking gel that overlaid a 10% acrylamide slab gel (1.00 mm thick). SDS slab gel electrophoresis was carried out for about 5 h at 25 mA/gel. The following proteins (Sigma Chemical Co., St. Louis, MO, USA) were used as molecular weight standards: myosin (220,000), phosphorylase A (94,000), catalase (60,000), actin (43,000), carbonic anhydrase (29,000) and lysozyme (14,000). These standards typically appear along the basic edge of the silver-stained 10% acrylamide slab gel [31] (Figures S2–S7 in Supplementary Material).

### 2.11. Computerized Comparisons

Duplicate gels were obtained from each sample. The gels were scanned with a laser densitometer (Model PDSI, Molecular Dynamics Inc., Sunnyvale, CA, USA). The scanner was checked for linearity prior to scanning with a calibrated Neutral Density Filter Set (Melles Griot, Irvine, CA, USA). The images were analyzed using Progenesis Same Spots software (version 4.5, 2011, Nonlinear Dynamics, Durham, NC, USA) and Progenesis PG240 software (version 2006, Nonlinear Dynamics, Durham, NC, USA). The general method of computerized analysis for these pairs included image warping followed by spot finding, background subtraction (average on boundary), matching, and quantification in conjunction with detailed manual checking. Spot % is equal to spot integrated density above background (volume) expressed as a percentage of total density above background of all spots measured. Difference was defined as fold-change of spot percentages.

### 2.12. Data Analysis

Data were presented as intensity levels at 547 spots for two replicates each of plasma samples representing Class I, II, III, and controls. The goal was to determine if there were spots, where infected patients differed significantly from healthy controls, and further if there were spots where I, II, and III differed from each other. The spot data for classes I, II, III, and, control (C), were transformed to the natural log scale to approximate a normal distribution. The 2 replicates at each spot for each group were denoted as *X_isg_* where, *i =* 1, 2; *s =* 1,*…*, 547; *g =* I, II, III, C. Then *l_isg_* = *ln(X_isg_)* is the log of this value. The mean of logs for this spot and group is:

(1) $$\bar{l_{sg}}=(log\;X_{1sg}+log\;X_{2sg})/2$$

Normalization for the control sample on this scale was done by subtracting $\bar{l_{sC}}$ rather than dividing by it, and normalized means on the log scale were defined as

(2) $$\Delta_{sg}=\bar{l_{isg}}-\bar{l_{isC}}$$

Geometric means are much more resistant to outlier effects, thus the geometric mean:

(3) $$e^{\bar{l_{sg}}}={\left(X_{1sg}X_{2sg}\right)}^{1/2}$$

for spot *s* and group *g* were used to determine the fold changes, *e^∆sg^*, between samples I, II, III and control C. The means of logs were standardized by normalizing to the median absolute deviation (MAD), normalized for unbiased estimation of σ for a normal distribution calculated from all *∆sg* values, to produce *p*-values. This method of normalization is resistant to extreme outliers. The unadjusted two-sided *p*-value for spot *s*, *s* = 1… max # of spots on each 2D gel (547 in our study) and group *g*, *g* = patient groups (I, II, and III) *vs.* controls, is

(4) psg=2Φ(−|Δsg/MAD|)

where, Φ is the standard normal cumulative distribution function (CDF). To control for the large number of multiple comparisons, sets of highly up- and down-regulated locations for each disease class were identified using Benjamini–Hochberg false discovery rate (FDR) analysis [32,33]. Briefly, to limit FDR to a desired level *q* (usually 0.05) the unadjusted *p*-values were ordered in ascending order: (*p*(1) ≤ *p*(2) ≤ *p*(3) ≤ … ≤ *p*(N) where N = 547; the maximum number of spots). Significant spots were determined by the following procedure: (a) Find the largest k such that *p*(k) ≤ *q*k/N; and (b) declare significant all spots where the *p*-values are less than or equal to *p*(k), *i.e.*, those corresponding to *p*(1), *p*(2), …, *p*(k). An FDR-adjusted p-value for ordered unadjusted *p*-value *p*(i) is N*p*(i)/i; Those values are compared to *q* and reported in the results. Because we could not assume that the 3 comparisons of I, II, III to controls were independent, the limit for significance of *q* was lowered from the typical 0.05 value to *q* = 0.05/3 = 0.0167 by applying a Bonferroni-like adjustment [32,33]. Calculations were performed using the statistical package R [34]. Several spots identified this way were selected for Matrix-Assisted Laser Desorption/Ionization (MALDI) mass spectrometry at Columbia University Core facility.

### 2.13. In-Gel Digestion of Proteins

Gel spots were transferred to clean tubes, water was added to completely hydrate gels, and the plastic coating was removed with clean tweezers. Gel spots were prepared for digestion by washing twice with 100 μL 0.05 M Tris, pH 8.5/30% acetonitrile for 20 min. with shaking, then with 100% acetonitrile for 1–2 min. After removing the washes, the gel pieces were dried for 30 min. in a Speed-Vac concentrator. Gels were digested by adding 0.08 μg modified trypsin (sequencing grade, Roche Molecular Biochemicals) in 13–15 μL 0.025 M Tris, pH 8.5. The tubes were placed in a heating block at 32° and left overnight. Peptides were extracted with 2 × 50 μL 50% acetonitrile/2% TFA; the combined extracts were dried and resuspended in matrix solution.

### 2.14. MALDI-MS Analysis

Matrix solution was prepared by making a 10 mg/mL solution of 4-hydroxy-α-cyanocinnamic acid in 50% acetonitrile/0.1% TFA and adding two internal standards, angiotensin and ACTH 7-38 peptide, to the matrix solution. The dried digest was dissolved in 3 μL matrix/standard solution and 0.5 μL was spotted onto the sample plate. When the spot was completely dried, it was washed twice with water. MALDI mass spectrometric analysis was performed on the digest using an Applied Biosystems Voyager DE Pro mass spectrometer in the linear mode.

### 2.15. Peptide Mass Search

Peptide masses were entered into search programs to search the NCBI and/or SwissProt databases for a protein match. Programs used are Mascot at www.matrixscience.com and MS-Fit at http://prospector.ucsf.edu. Cysteine residues were modified by acrylamide during electrophoresis. Error tolerance was set at 0.5 Da for average masses.

### 2.16. PAI-1 Expression in HCPS Samples

Active Human PAI-1 functional assay (#HPAIKT) and Human PAI-1 total antigen assay (#HPAIKT-TOT) ELISA kits were used to measure the expression of PAI-1 in HCPS samples, following protocols supplied by the manufacturer (Molecular Innovations, Novi, MI, USA).

## 3. Results and Discussion

### 3.1. Cell Barrier Dysfunction Is Induced by Thrombin and Other Factors Present in HCPS Patient Plasma Samples

**Figure 1:**
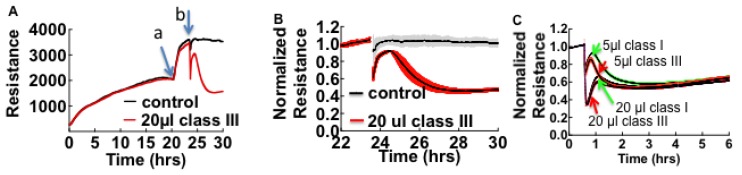
Effect of pooled (*n* = 4) class III Hantavirus Cardiopulmonary Syndrome (HCPS) patient plasma on cell barrier properties of Vero E6 cells measured by Electric Cell-substrate Impedance Sensing (ECIS). (**A**) Vero E6 cells were plated in electrode-containing dishes at 10^5^ cells/cm^2^ and allowed to attach, spread, and organize for at least 20 h. Cellular impedance was measured continuously at a single frequency of 4000 Hz. Increase in resistance corresponds to increasing cell barrier function. a: The rapid increase in resistance at about 20 h occurred after the media was exchanged; b: Class III plasma collected on day of admission and healthy control plasma were added to monolayers; (**B**) Comparing the effect of patient plasma and controls by normalizing data from (**A**), at baseline established after media was refreshed. Error bars represent duplicate measurements; (**C**) Effect of 4-fold dilution on the barrier function of confluent monolayers of pooled plasma from classes I, and III patients. Data also show that the loss of resistance is caused by a concentration-dependent fast acting component and a slowly acting component, which is relatively insensitive to dilution.

When cells are exposed to HCPS patient plasma, the accompanying changes in cell morphology such as loss of cell barrier function are measured as drops in resistance over time. Vero cells propagate rapidly and form confluent monolayers characterized by high resistance within 24 h compared to endothelial cells (Figure S1 in Supplementary Material). Figure 1 shows a prototypical experiment where, Vero E6 were allowed to attach, spread, and organize in 200 µL of cell media in ECIS cultureware wells for at least 20 h while resistance was measured continuously.

Cell culture media was refreshed and cells were allowed to equilibrate for at least two hours before 20 µL volumes of pooled plasma from class III HCPS and healthy subjects were added to 200 µL of media in the wells (Figure 1A). To compare resistance measurements of monolayers treated with healthy control and HCPS samples, data were normalized to the baseline plateau reading taken after media was changed (Figure 1B). Exposure of confluent cell monolayers to HCPS patient plasma causes an immediate drop in resistance and a partial recovery. The drop in resistance reflected the changes in cell morphology due to loss of cell barrier function [23,24]. In contrast, samples from healthy controls did not induce significant changes to monolayer resistance as shown in Figure 1B. During the course of studies we also found out that apparently healthy subjects with non-specific inflammatory states as measured by a Complete Blood Count with Differential [35,36], elicited negative change in resistance in ECIS assays. Such samples were excluded as controls. To determine whether ECIS could be used to distinguish between classes I, II, and, III HCPS, different class samples were pooled (*n = 5 for class I and II and n = 4 for class III*) and analyzed with ECIS. When used at 20 µL the three classes caused comparable changes in resistance over the time course. However, in diluted samples, at least two types of edemagenic factors that gave transient and prolonged changes in resistance were revealed as components of the HCPS plasma (Figure 1C).

**Figure 2:**
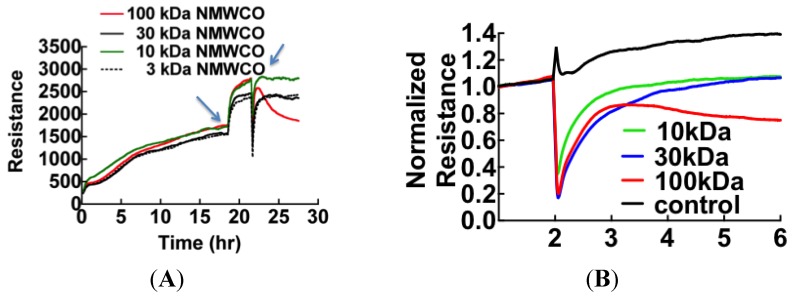
ECIS measurement of molecular weight-based fractions of patient plasma showing that proteins retained by 100 kDa cutoff filters cause permanent loss of resistance. Arrows indicate the time points when media was changed (19 h) and plasma samples were added (22 h). (**B**) Baseline normalized ECIS profiles shows the effect of plasma proteins fractionated by molecular weight on cell barrier function (in different sample from (**A**)). Component protein(s) retained by the 100 kDa filter are shown to cause sustained loss of resistance, while lower molecular weight proteins of 30 kDa and 10 kDa range are shown to show reversible loss of resistance.

The first step to identify the edemagenic factors was to fractionate patient plasma with graded microcon centrifugal filters (Millipore, Danvers, MA, USA) with the descending nominal molecular weight cutoff (NMWCO) limits of 100 kDa, 30 kDa, 10 kDa and 3 kDa. Because NMWCO limits are based on steric bulk estimates, rather than true molecular weight, it is useful to extend the cutoff to half the suggested filter grade. The retentate from each filter was added in the original volume to ECIS wells (Figure 2). Retentates of the 100 kDa filter caused the loss in cell barrier dysfunction similar to thrombin [37,38] while the eluates induced reversible loss of resistance (Figure 2B).

Thrombin and plasmin are central enzymes involved in coagulation and fibrinolysis, respectively. Thrombin is a coagulation protease that is generated at sites of vascular injury and converts fibrinogen into fibrin, thus forming clots [39,40,41,42,43,44,45]. Fibrinolysis, the breakdown of fibrin blood clots, involves the activation of plasminogen to plasmin and its subsequent proteolysis of clots, protects against thrombotic occlusion of the host’s arterial and venous systems and is necessary for normal wound healing [46,47,48,49]. Proteolytic activity of both enzymes can lead to loss of cell barrier function [42,50,51,52,53,54]. While SNV infections are not typically associated with significant alterations in hemostasis, thrombocytopenia and disseminated intravascular coagulation have been noted in SNV infections [9,55,56].

**Figure 3:**
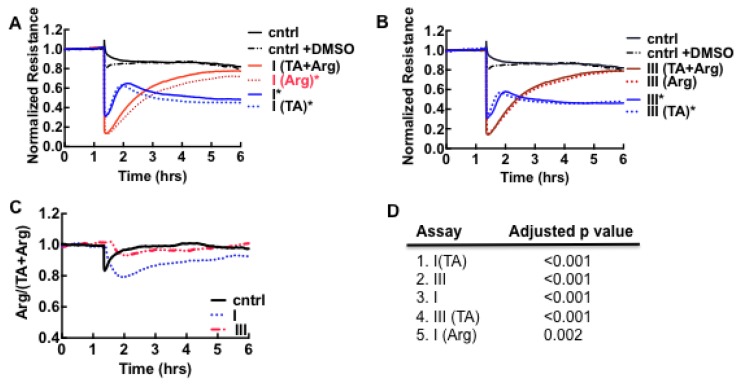
Relative effects of pooled classes I and III plasma samples on the barrier function of Vero E6 cell monolayers in assays using thrombin (Arg) and plasmin (TA) inhibitors. (**A**) Class I and controls (cntrl); (**B**) Class III and controls (cntrl); (**C**) Ratiometric plot of *Arg/(Arg + TA) versus* time, used to determine activity of plasmin in classes I and III samples. The result shows that plasmin expression is significant in class I. Dimethyl sulfoxide (DMSO) was used to solubilize argatroban. The final concentration of DMSO in media was uniformly kept at <1% in all samples; (**D**) List of false discovery rate (FDR) - adjusted *p*-values (<0.01) used to determine whether the recovery endpoint (6 h) of cell barrier function in the assay conditions shown in A and B differ significantly from the controls. The assays on the list were found to differ significantly from the controls.

To determine whether hemostatic impairment was a significant factor in our cohort, samples were treated with argatroban [41] or hirudin [57], drugs that inhibit thrombin activity. Tranexamic acid [58], a synthetic analog of the amino acid lysine that inhibits activation of plasminogen to plasmin and blocks plasmin binding to fibrin, was administered separately or together with argatroban, to the samples prior to the ECIS assay. The data shown in Figure 3A,B suggest that loss of resistance was largely due to thrombin in day 0 samples. Samples treated with argatroban (*Arg* in Figure 3) or both inhibitors (*TA + Arg*) in Figure 3 recovered to control levels after a few hours whereas tranexamic acid-treated samples (*TA* in Figure 3) retained a profile close to the untreated samples (samples I and III in Figure 3). Thrombin induces cytoskeletal remodeling characterized by the activation of the small GTPase, RhoA associated with stress fibers and loss of cell barrier function by activating the family of protease-activated receptors (PAR-1, PAR-3 and PAR-4)-expressed on platelets and a variety of cell types [39,40,41,42,43]. Plasmin is known to activate PAR-1 and PAR-4 [59]. Because thrombin is an enzyme, rather than a ligand agonist, one thrombin molecule can cleave and activate several of the cognate protease-activated receptors (PAR-1–4), thus classes I and III appear to elicit the same level of thrombin-mediated response in ECIS [38,60,61].

The recovery kinetics and endpoint of *Arg*-only samples appeared to diverge from *TA + Arg* in class I but overlapped in class III assays. We then calculated the ratio of the thrombin-inhibited samples (*Arg-only*) and thrombin- and plasmin-inhibited (*TA + Arg*) assays in order to determine whether the assay results diverged significantly. As shown, in Figure 3C the average time course result for the ratiometric calculation *Arg/(TA + Arg)* was determined as unity, for control and class III, but less than one for class I. The differences between the recovery endpoint for each assay condition in Figure 3A,B and the controls were analyzed for statistical significance using False Discovery Rate [32] (see methods). The results in Figure 3D are consistent with the ratiometric analysis, that plasmin expression is significant in class I but not measurable by ECIS in class III. The apparent deficit in plasmin activity in class III, which suggests fibronolytic-impairment was subsequently confirmed by proteomic analysis of the plasma as described below.

**Figure 4:**
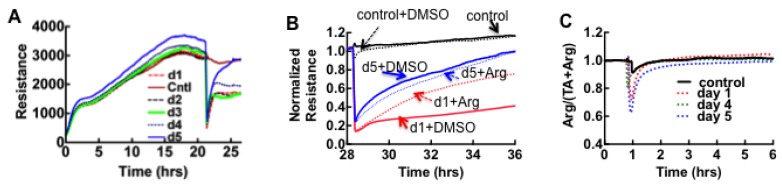
ECIS analysis of cell monolayers exposed to serial plasma samples. (**A**) Serial changes of ECIS profiles on different days suggest the diminution of thrombin expression on day five; (**B**) Relative effect of argatroban on day one and day five samples, implicate thrombin expression in day 1 sample and negligible amount on day five sample, perhaps indicating patient recovery. Dimethyl sulfoxide (DMSO) was used to solubilize argatroban. (**C**) Ratiometric plot of *Arg/(Arg+TA) versus* time was used to determine activity of plasmin in class II samples. The results show that plasmin activity is not significant in class II as measured by assay endpoint determinations between controls and class II serial samples.

Serial classes II and III samples collected on admission and up to five consecutive days thereafter were examined by ECIS to track the dual expression of thrombin and plasmin during this period. There were no significant changes between admission day and serial collections of class III samples. The ECIS results for pooled class II samples collected over the first four days were comparable to the thrombin-dominated results of classes I and III samples, whereas day five was comparable to argatroban-treated samples as shown in Figure 4A,B. The ratiometric profile (*Arg/(TA + Arg)*) of the serial samples in the class II cohort revealed no significant plasmin activity (measured at the 6 h time point) in all samples (Figure 4C).

The most interesting finding from the class II cohort is the overt diminution of thrombin expression on day five. This suggests that subclinical changes in hemostasis are a component of HCPS caused by SNV as further discussed below.

To summarize this section, we have established Vero E6 cells as a sensing platform to assess subclinical changes in hemostatic balance due to SNV infection. We used Vero E6 cells, because of their capacity to proliferate readily in culture, compared to endothelial cells. Our study shows that thrombin confers the same barrier weakening effect on epithelioid Vero E6 cells as endothelial cells (Figure S1), in contrast to the opposite effect previously shown in A549 cells [62]. Thus our finding suggests that thrombin can elicit differential responses based on cell type within the same lineage. Although much earlier studies of SNV-induced HCPS have documented coagulation abnormalities [55], they have generally not been considered to be a significant component of HCPS pathophysiology [56,63]. A ratiometric analysis of samples treated with thrombin and thrombin + plasmin inhibitors can be used to capture the subtle changes in hemostatic balance that are induced by SNV infection. The apparent diminution of plasmin expression associated with disease severity (class I *vs.* class III) in the ECIS analysis portends a prothrombotic state, which is shown below to be due to very high levels of plasminogen activator inhibitor type 1 (PAI-1) [18,64,65,66,67,68] expressed in class III patients.

### 3.2. Sin Nombre Virus Disrupts Cell Barrier Function Vero E6 and Endothelial Cells in Vitro

**Figure 5:**
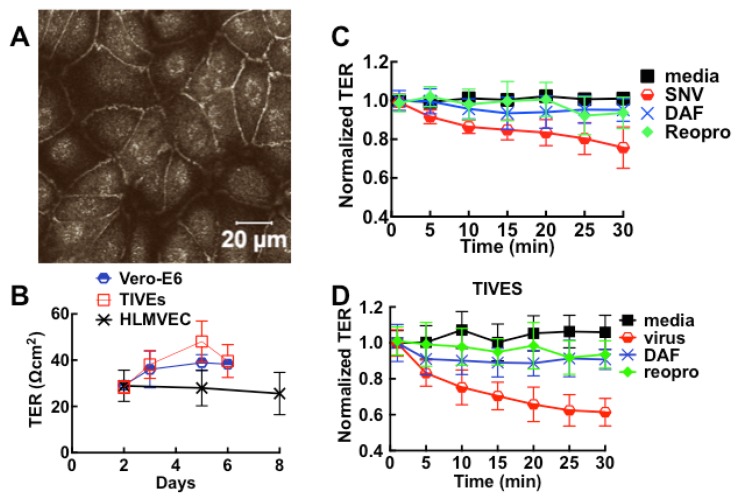
SNV induces loss of cell barrier function in intact tight-junction forming monolayers of polarized Vero and TIVE. (**A**) Confocal images of tight junctions in polarized monolayers; (**B**) Development of tight junctions in polarized monolayers grown in 6 mm microporous filter inserts at 150,000 cells/well was measured on days shown in the plot of TER *versus* time for HLMVEC, TIVE and Vero E6 cells. The error bars are standard deviations from 12 monolayers; (**C**, **D**) Effect of live SNV on the barrier integrity of Vero E6 and TIVE monolayers under different conditions described in the text. Error bars represent standard deviations from independent replicates of 3 monolayers.

Hantavirus infection has been shown to contribute to the disruption of cell barrier function *in vitro* [15,69,70,71]. We therefore exposed confluent monolayers to live virus to determine whether the interaction between host cells and SNV contributes to loss of cell barrier function. For cells grown to confluence in 8-well Lab-Tek chambers plates or transwell filter supports in 24 well plates, cell adhesion to the extracellular matrix and neighboring cells provides extrinsic cues [72] to polarize and form tight junctions (Figure 5A) manifested by an increase in transmonolayer electrical resistance (TER) measurements (Figure 5B). As in ECIS measurements, increase in TER correlates with development of tight junctions, while a decrease in TER indicates a disruption in the integrity of cell barrier function. Addition of virus inocula (moi 0.1) induced drops in cell barrier function in Vero E6 and endothelial cells, with endothelial cells undergoing a 20% greater loss in cell barrier function compared to Vero E6 cells. Cell barrier function was significantly conserved in samples when cognate interaction between SNV and cells was blocked with soluble DAF (sDAF), or Reopro (Figure 5C,D). Inhibitor-treated cells were also less susceptible to infection as previously noted [25,26,73,74,75].

We do not believe that the plasma samples used in this study contained significant quantity of intact virions capable of interaction with cells for the following reasons: First, even though viral S-segment RNA proteins have been found in our samples and elsewhere [14], it is likely that the viral RNA is not associated with intact particles. Because hantavirus particles bind to DAF with high affinity [25], it is probable that the virions were attached to erythrocytes and other DAF-bearing cells when the plasma was separated from whole blood. Second, the presence of high titers of neutralizing antibodies would inhibit virus from engaging cellular receptors [14]. Third, we used a seven-day focus assay to test for the presence of infectious virions in our samples and from the assay results these samples were free of replication-competent particles. Nevertheless, our data here show that SNV causes loss of cell barrier function, which our earlier study with killed UV-SNV particles implicated a mechanism that involved the activation of RhoGTPases such as Rac1, Rap1 and RhoA [76]. For the purpose of this study, this finding suggests that cognate interaction between virus particles and host cells is one of several factors that contribute to the loss of cell barrier function in HCPS.

### 3.3. Immunological Response to Hantavirus Infection

**Figure 6:**
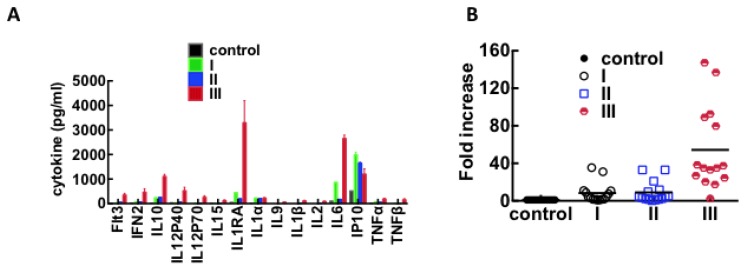
Plot of cytokines that are differentially upregulated in plasma from classes I, II, and III HPCS patients. (**A**) Duplicate samples of pooled plasma (day 0, *n* = 5 for classes I and II; *n* = 4 for class III) were analyzed using a Millipore HCYTMAG-60K-PX41 Cytokine kit. Significant increases in cytokine expression were determined by a two-way ANOVA with a Bonferroni's multiple comparison test; (**B**) The data from (**A**) were recast as fold increase above normal expression levels.

We used a 41plex HCYTMAG-60K-PX41 Cytokine kit (Millipore, Danvers, MA, USA) to identify differentially-upregulated cytokines in pooled plasma samples from classes I, II, and, III patients. Patient samples (I, II, and III) and controls were analyzed in duplicate. The data were transformed to natural log scale to approximate normal distribution and then normalized to controls as described in methods for 2D gels. Non-parametric Anova with Bonferroni multiple comparison tests at 99% confidence was used to determine whether the increase in cytokine expression in all classes of HCPS samples was significant relative to healthy controls. 15 of the 41 measured cytokines were significantly upregulated largely in class III in accordance with prior studies [11,13,56,77,78,79,80]. The data are summarized in Table 1 and Figure 6.

**Table 1:** List of significantly upregulated cytokines from Bonferroni’s multiple comparisons test (α = 0.01) of 41 cytokines measured in plasma samples from classes I, II, and III HCPS patients using a HCYTMAG-60K-PX41 Cytokine kit.

Cytokine	Adjusted *p*-Value
**Flt3**	
control *vs.* II	0.0002
control *vs.* III	<0.0001
**IFN2**	
control *vs.* III	<0.0001
**IL10**	
control *vs.* I	<0.0001
control *vs.* II	<0.0001
control *vs.* III	<0.0001
**IL12P40**	
control *vs.* III	<0.0001
**IL12P70**	
control *vs.* III	<0.0001
**IL15**	
control *vs.* III	0.0029
**IL1RA**	
control *vs.* III	<0.0001
**IL1α**	
control *vs.* I	<0.0001
control *vs.* II	<0.0001
control *vs.* III	<0.0001
**IL9**	
control *vs.* III	0.0003
**IL1β**	
control *vs.* III	<0.0001
**IL2**	
control *vs.* III	<0.0001
**IL6**	
control *vs.* III	<0.0001
**IP10**	
control *vs.* I	<0.0001
control *vs.* II	<0.0001
control *vs.* III	0.0003
**TNFα**	
control *vs.* III	<0.0001
**TNFβ**	
control *vs.* III	<0.0001

The global increase of cytokine expression in class III patients comprises some essential elements of the presumptive “cytokine storm” associated with HCPS pathogenesis [2,9,56]. It is also worth noting that some HCPS pathogenesis models have linked cell barrier dysfunction to increased expression of vascular endothelial growth factor (VEGF) [69,70,71]. Herein and elsewhere, normal plasma levels of VEGF have been measured in mild and severe HCPS subjects [81] or animal models [82] but have been shown to be elevated in pulmonary edema fluid [81]. Our results are consistent with the idea that VEGF does not cause systemic edemagenic effects upon release in circulation plasma due to dilution [83], and therefore its putative effects are localized at sites of expression.

The small size of our study cohort benefits from a previous examination of a broader HCPS patient cohort admitted to UNMH [84,85]. Our study adds a new level of molecular basis to the pathogenesis of HCPS that parallels some of the clinical features previously found to distinguish surviving and terminal patients. For example, we are thus able to discuss the elevated inflammatory mediators in class III patients relative to the survivor classes, in the context of earlier work [86]. The inflammatory mediators that we found to be significantly elevated, are among the cytokines (IL-1α, IL-1β, IL-6, TNF-α, IFN-γ, IL-2, IL-4 and TNFβ) detected in monocytes/microphages and activated T cells, that extravasated into lungs and spleen tissues of fatal HCPS cases [86]. A previous analysis of hantavirus load in the plasma of HCPS patients admitted to UNMH, found that viral RNA copy levels were ten times higher in fatal cases than in survivors [14]. Elsewhere, it has been suggested, a robust pro-inflammatory cytokine response is required for virus clearance [2,9,87,88]. The concomitant increase in pro-inflammatory and anti-inflammatory cytokines in class III would suggest increased vascular damage in this class compared to classes II and I.

### 3.4. Plasma Protein Profiles of HCPS Patients by 2D Gel Analysis Show that the Largest Differential Changes in Protein Expression Occurred in Class III Patients

Duplicate patient samples (I, II, and, III) *versus* controls were analyzed in terms of readouts from a laser densitometer and computer analyzed for differential expression of proteins as measured by spotsize in the patient plasma relative to control (Figures S2–S7 in Supplementary material). Computer generated analysis was also confirmed by visual inspection to detect outliers.

**Figure 7:**
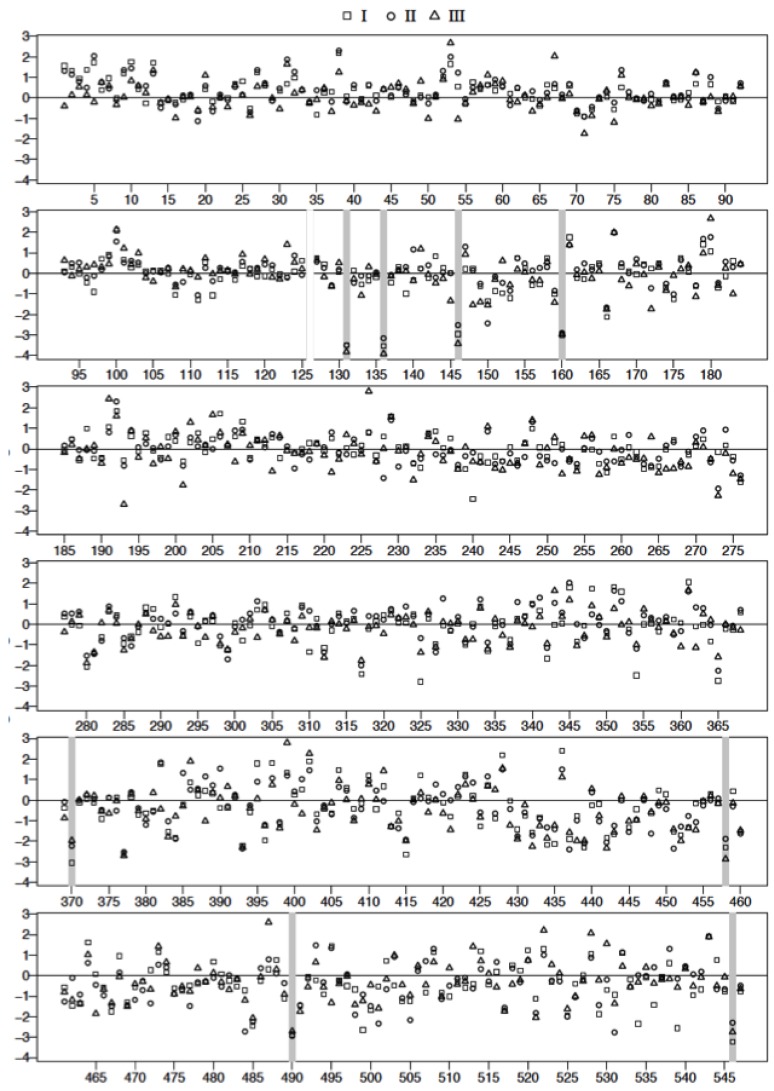
Plot of the logarithm of geometric mean fold change relative to control, (($\Delta_{sg}=\bar{l_{isg}}-\bar{l_{isC}}$) see text for details) for all 2D gel spots derived from the analysis of Classes I, II, and III plasma. Spot reference numbers are on the horizontal axis, and there is one value for each of the three classes. The spots marked with a grey bar represent ones associated with statistically significant (False Discovery Rate) changes relative to control. Spot 126 was rejected as an outlier after visual inspection of the gel spots.

**Figure 8:**
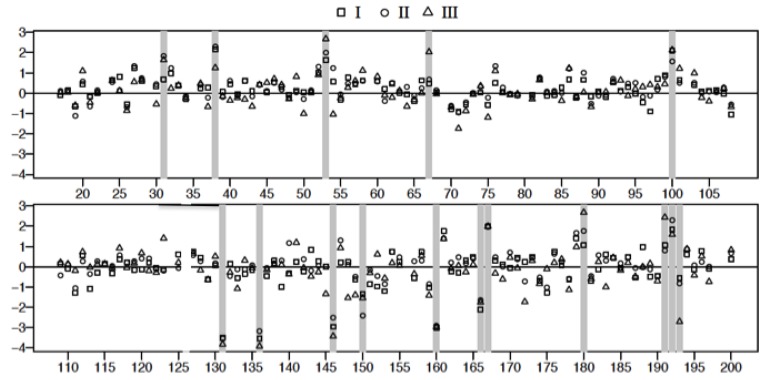
Plot of the logarithm of geometric mean fold change relative to control, (($\Delta_{sg}=\bar{l_{isg}}-\bar{l_{isC}}$) see text for details) for 2D gel spots associated with proteins above the nominal molecular weight of 50 kDa derived from the analysis of Classes I, II, and III plasma. Spot reference numbers are on the horizontal axis, and there is one value for each of the three classes. The spots marked with a grey bar represent spots associated with statistically significant changes (FDR) relative to control.

The spot intensity data were transformed to the natural log scale to approximate normal distribution. The logs of geometric means of duplicate data sets representing fold changes in the expression of proteins relative to controls (547 spots/classification) were plotted as shown in Figure 7. Spot data showing significant differences between patient samples and controls were determined from adjusted *p*-values using FDR (and Bonferroni) analysis [32,33] as outlined in the methods. Adjusted *p*-values were calculated following two different conditions where: all data spots were considered and only those spots associated with molecular weight exceeding 50 kDa in accordance with our fractionation experiments (Figure 2B). The natural log transformed raw data are shown in Figure 7 and Figure 8, respectively. The results of the FDR analysis using 547 spots and 200 spots in the ≥ 50,000 kDa group are shown in Table 2 and Table 3, respectively. Only 8 spots out of 547 spots per disease group classification, compared to 16 spots (out of 200 per disease group classification) in the ≥50,000 kDa group, were declared to be statistically significant (see methods).

**Table 2:** List of FDR (Benjamin-Hochberg False Discovery Rate) adjusted *p*-values that were less than 0.05/3 using all 547 spots in each group. The assumed co-dependence of the three groups (I, II, and III) prompted the Bonferroni-like adjustment to enhance the criterion for significance to 0.0167 rather than the customary 0.05. ∆ fold values relative to C are determined by dividing the geometric mean of each group by the geometric mean of C. Fold increases less than one are reported as geometric mean C/geometric mean group. Blank entries in the table represent FDR adjusted *p*-values > 0.0167.

	Spot	FDR Adjusted *p*-Value I *vs.* C	FDR Adjusted *p*-Value II *vs.* C	FDR Adjusted *p*-Value III *vs.* C	Mol. Wt	∆ Fold I	∆ Fold II	∆ Fold III
1	131	0.001	0.001	0.000	62,450	−33.0	−34.2	−45.7
2	136	0.001	0.006	0.000	59,941	−34.2	−24.2	−50.2
3	146	0.009		0.001	55,874	−19.2	−12.5	−30.6
4	160	0.008	0.016	0.012	54,420	−20.3	−18.2	−19.4
5	370	0.007			26,948	−21.2	−9.4	−7.2
6	458			0.016	19,388	−9.9	−6.7	−17.7
7	490	0.012	0.016		16,089	−17.5	−19.1	−15.0
8	546	0.003			6744	−25.3	−9.9	−15.5

**Table 3:** List of FDR adjusted *p*-values < 0.0167 for spots with molecular weight >50,000 Da. ∆ fold values are relative to C and were determined by dividing the geometric mean of each group by the geometric mean of C. Fold increases less than 1 are reported as geometric mean C/geometric mean group. Blank entries in the table represent *q*-values > 0.0167.

	Spot	FDR Adjusted *p*-Value I *vs.* C	FDR Adjusted *p*-Value II *vs.* C	FDR Adjusted *p*-Value III *vs.* C	Mol. Wt	∆ Fold I	∆ Fold II	∆ Fold III
1	31		0.016		170,054	2.0	6.3	5.2
2	38	0.003	0.001		149,443	8.7	9.9	3.5
3	53		0.007	0.000	117,167	5.1	7.4	14.5
4	67			0.004	92,464	1.6	1.9	7.6
5	100	0.004		0.003	71,422	8.2	4.8	8.4
6	131	0.000	0.000	0.000	62,450	−33.0	−34.2	−45.7
7	136	0.000	0.000	0.000	59,941	−34.2	−24.2	−50.2
8	146	0.000	0.000	0.000	55,874	−19.2	−12.5	−30.6
9	150		0.000		54,232	−3.8	−11.4	−4.7
10	160	0.000	0.000	0.000	54,420	−20.3	−18.2	−19.4
11	166	0.004			52,793	−8.3	−5.3	−5.8
12	167	0.007	0.007	0.005	55,228	7.3	7.2	7.3
**13**	**180**			**0.005**	**54,005**	**2.9**	**5.8**	**14.5**
14	191			0.000	52,322	2.9	2.2	11.4
15	192		0.001		50,890	6.3	9.8	4.9
16	193			0.000	52,090	−1.7	−2.3	−14.9

### 3.5. Mass Spectrometric Analysis Shows that Prothrombotic Factors Are Significantly Upregulated in Class III Patients

The spots that we determined to be up- or down-regulated significantly, were excised and in gel-digested with trypsin followed by mass spectrometric analysis (Table 4). The sequence coverage was 5%–52% for all spots except spot #53, elongation growth factor 2, which was identified by LC-MS/MS (Supplementary Tables S1–S19). The list of up- and down-regulated proteins and their biological functions are listed in Table 4. The overexpressed proteins are; pregnancy zone protein, protease bound α-1-antitrypsin, elongation factor 2, hemopexin, prothrombin, C3 complement component, plasminogen activator inhibitor 1, isocitrate dehydrogenase, and haptoglobin. Among the depleted proteins are, α-1-antitrypsin (uncleaved), complement component C4B, carboxypeptidase N catalytic chain, chain B complement C3C, and apolipoprotein A-1.

**Table 4:** List of significant FDR-selected spots (*cf.* Table 2 and Table 3) from difference 2D gels identified by mass spectrometry. Functional activity of proteins was identified using Gene Ontology analysis (http://geneontology.org).

Spot #	Name	UniProt or NCBI Accession	Biological Function
(+) 31	pregnancy zone protein	P20742	a
(+) 38	α-1-antitrypsin	P01009	b
(+) 53	elongation factor 2	EF2	c
(+) 67	prothrombin/hemopexin	P00734/P02790	d
(+) 100	6-phosphofructo-2-kinase/fructose-2,6-bisphosphatase	Q16877	e
(−) 131, 136,146,160	α-1-antitrypsin	P01009	f
(−) 150,166	complement component C4B	gi:187771	g
(+) 167	C3 complement component	gi:194384410	h
(+) 180	plasminogen activator inhibitor	P05121	i
(+) 191	isocitrate dehydrogenase	O75874	j
(+) 192	haptoglobin	P00738	k
(−) 193	carboxypeptidase N catalytic chain	P15169	l
(−) 370	chain B, complement C3C	gi:78101270	m
(−) 458, 490	apolipoprotein A-1	P02647	n

a: *Pregnancy zone protein (PZP)* is a proteinase inhibitor that is normally upregulated during pregnancy. PZP is believed to inhibit fibrinolytic activity binding to tissue plasminogen activator (tPA), a serine proteinase that catalyzes plasmin formation [89,90]; b,f: *α-1-antitrypsin* is an acute phase protein that inhibits serine proteases. Its primary target is elastase, but it also has a moderate affinity for plasmin and thrombin. Irreversibly inhibits trypsin, chymotrypsin and plasminogen activator; c: *Elongation factor 2* is a mediator of protein synthesis; d: (1) *Prothrombin* is an inactive precursor of thrombin that is converted to the latter by the proteolytic activity of the enzyme Factor X; (2) Hemopexin antiflammatory mediator [91,92]; e: *6-phosphofructo-2-kinase/fructose-2,6-bisphosphatase* is an enzyme that is important for carbohydrate metabolism; g,h,m: *Complement* system components; i: *Plasminogen activator inhibitor-1 (PAI-1)* is a regulator of plasmin-mediated pericellular proteolysis in the vasculature [16,17,18] Elevated expression of PAI-1 is generally associated with impaired fibrinolytic function and thrombosis[65]; j: *isocitrate dehydrogenase*. Mitochondrial enzyme involved cellular energy production that plays a role in the protection of cells from reactive oxygen species; k: *haptoglobin* Binds to free plasma hemoglobin and enables recycling of heme iron which prevents kidney damage. Haptoglobin is involved in modulating acute phase response and additionally has antimicrobial and antioxidant properties; l: *Carboxypeptidase N catalytic chain (CPN1)* regulates the activity of vasoactive peptide hormones, growth factors and cytokines by specifically cleaving the *C*-terminal basic residues from the substrates; n: apolipoprotein A-1 is involved in lipid metabolism, is also believed to mediate infection by Dengue virus. Its levels are known to decrease during infection.

In some cases we observed the down regulation of native and the upregulation of their reacted forms. For example, α-1-antitrypsin (MW = 50–67 kDa; spot #s 131, 136, 146, 160) is an acute phase proteinase inhibitor, which targets the active site of neutrophil elastase [93]. Its activity is governed by its covalent attachment to the substrate: [α-1-antitrypsin] + [substrate] → [α-1-antitrypsin•substrate]. Thus, [α-1-antitrypsin•substrate] (spot #38 MW = 149,443) was identified as an upregulated component in the 2D gel, while the circulating [α-1-antitrypsin] native form was depleted incrementally from classes I to III. It is likely that depletion of α-1-antitrypsin allows neutrophil elastase to disrupt connective tissue in lungs (elastin), which alters the elasticity of the lungs, thus may contribute to respiratory complications that is a hallmark of HCPS. Complement activity is indicated by the upregulation of C3 activation products (spot #167) and consumption of C3c and C4b (#150, 166, and 370).

### 3.6. Acute Derangement of PAI-1 Expression in All Class III Patients Preceded Death

**Figure 9:**
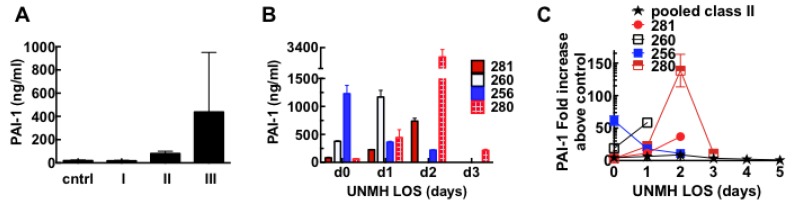
Plasminogen activator inhibitor 1 (PAI-1) is highly upregulated in Class III HCPS patients as measured with ELISA assays. (**A**) Average expression of PAI-1 samples from individual control, cntrl (*n* = 5), Class I (*n* = 5), II (*n* = 5), and III (*n* = 4) subjects. Data show, analysis results of samples collected on day of admission to UNMH. Error bars reflect subject-to-subject variability with median, upper and lower limits as follows: cntrl (19.1, 29.8, 12.6); I (17.6, 25.4, 14.1); II (82.8, 120.3, 55.8); III (230.5, 1377.0, 59.0); (**B**) Plot of active PAI-1 expression in individual class III subjects identified as 281, 260, 256 and 280. Sample collection ended on different days due to death of the subjects. Error bars are duplicate measurements; (**C**) Plot of fold increase in active PAI-1 measured in pooled samples of Class II and individual Class III HCPS patients *versus* length of hospital stay (LOS). Error bars are duplicate measurements.

Elevated expression of the serine protease inhibitor plasminogen activator inhibitor 1 (PAI-1) (spot #180) is generally associated with impaired fibrinolytic function and thrombosis [65]. The expression of active PAI-1 in HCPS patients was quantitatively confirmed by ELISA assays (Molecular Innovations), the results are summarized in Figure 9. We assayed active PAI-1 expression levels in individual patient admission day samples (day 0) in each severity group. As shown in Figure 9A, class I samples were of comparable magnitude to the controls, whereas the median value for class II was about four times as high as the control samples. PAI-1 expression in class III patients spanned a broad range (Figure 9A) of active PAI-1 expression. Examination of the individual class III samples over the duration of their hospitalization suggests that class III patients are predisposed to acute and extreme elevation of PAI-1 which was followed by death within 48 h, while the aggregate PAI-1 expression in the class II cohort did not exceed a level above a 10-fold increase which decreased to normal levels by day 5 (Figure 9C). The expression level of PAI-1 in class III subjects varied among subjects on day of admission and comparable in magnitude with class II cohort depending on disease severity at the time (Figure 9A). It is useful to describe the time course of PAI-1 expression together with their abstracted illness history and limited review of systems.

*256:* Patient was admitted to UNMH about 14 days after suspected contact with deer mice. Upon admission, the patient met all UNMH diagnostic criteria for severe HCPS [19,94] including pulmonary infiltrates, respiratory distress, nausea, fever, white blood cells (WBC) 23.9 × 10^3^/mL); platelets 17 × 10^3^/mL). Coagulation Parameters on day 0 were as follows: Prothrombin time (PT) 19.6 (reference range 9.9–13.9 s), activated partial thromboplastin time (aPTT) 54 s (normal range 24–37 s), international normalized ratio (INR) 1.87 (normal reference range <1.3), and fibrinogen 233 mg/dL (normal range 200–400 mg/dL). The longer PT and aPTT times suggest consumption of coagulation factors, and have been associated with death in SNV infections [55]. As our study samples were originally collected in EDTA tubes (instead of citrate) [95] it was not possible for us to examine the subsequent samples for serial changes in coagulation factors including d-dimers. For this patient, active PAI-1 was 50 fold above control samples on day of admission and dropped on subsequent days before the patient died. Thus the profile fits a subject whose disease state was well advanced on admission to UNMH.

*260:* Admitted to UNMH two weeks after suspected exposure to SNV with high fever, WBC 33K, platelets 115–116 × 10^3^/mL and very ill on day 0. Platelets remained high between 90 and 110 × 10^3^/mL on day one; however, patient died late on the same day. Our analysis found that active PAI-1 was 15 fold above normal on day 0 and then increased to over 50 fold above normal on day one. Without other knowledge about potential co-morbidity or differential diagnosis, the presentation of relatively high platelets is unusual for a class III case. However the high levels of PAI-1 are consistent with the characteristics of the class III cohort.

*280:* Formally very healthy patient was transferred to UNMH with flu-like symptoms and suspicion of HCPS. Consistent with this PAI-1 levels measured in day 0 samples were only two fold above normal range. Platelet levels were read at 15 × 10^3^/mL, and 26 × 10^3^/mL on day one and two, respectively. PAI-1 levels increased up to over 15 and 100 fold, respectively, and dropped down to 10 fold on day three when the patient died.

*281:* Patient was transferred to UNMH on suspicion of exposure to deer mice. HCPS was confirmed by serological detection of antibodies against viral N proteins and glycoproteins [19,94]. No signs of acute distress on admission, WBC 8K, platelets 31 × 10^3^/mL with coagulation parameters: PT = 14.2 s, INR = 1.06, aPPT = 41 s, elevated fibrinogen 525 mg/dL. On day 1 patient exhibited worse symptoms of HCPS as WBC increased to 10.5 × 10^3^/mL, platelets dropped to 18 × 10^3^/mL. Patient died late on day 2. PAI-1 levels on day 0 were two-fold above normal, increased to about 10 fold and 30 fold on day one and two, respectively, as HCPS symptoms worsened. There was no report of serial measurements of coagulation factors in the abstract.

It is worth noting that a better understanding of the vascular thombogenesis in these patients could have been possible with a clear picture of the patients’ medical record. We do not know the day when ECMO was administered for each patient (classes II and III), due to differences in severity at admission. As all ECMO patients are subject to anticoagulant treatment (heparin), the PAI-1 results could have been attenuated by medical treatment. Nevertheless our results establish a framework for better understanding of SNV pathogenesis as well as instituting critical parameters for a future prospective study of the disease. The significant finding here is that the lack of plasmin activity that is apparent in the ECIS assays (classes II and III) is consistent with the inhibition of plasminogen activation by the elevated levels of PAI-1 and pregnancy zone protein (spot #31) in these patients (*cf*. Figure 3 and Figure 4).

### 3.7. Model of Factors Influencing PAI-1 Upregulation

**Figure 10:**
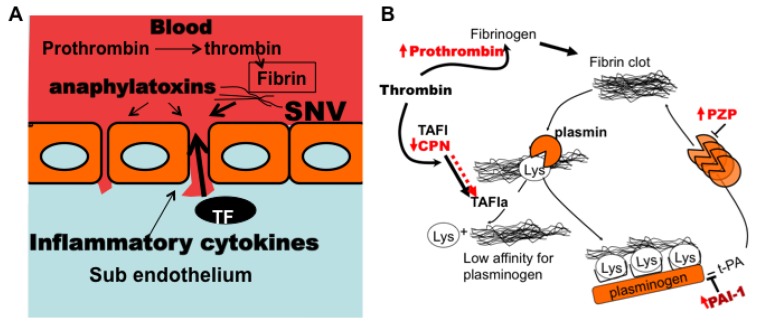
Model of hemostatic impairment and adverse PAI-1 generation in class III HCPS patients. (**A**) Vascular permeability in HCPS is induced by viral engagement to endothelial cells as well as the severe inflammatory insult from cytokines and complement anaphylatoxins. Damage to the endothelial wall, and subsequent efflux of plasma into the interstitium triggers thrombogenesis as subendothelial tissue factor is exposed to coagulation factors; (**B**) Local accumulation of fibrin thrombi develops profibrinolytic activity in order to prevent thrombosis. Robust antifibrinolytic activity is upregulated in class III HCPS. Carboxypeptidases, such as carboxypeptidase N (CPN) and activated thrombin-activable fibrinolysis inhibitor (TAFIa) play important roles in controlling fibrinolysis. Plasmin degradation of fibrin is enabled by the exposure of *C*-terminal lysine residues (Lys), to which it binds. Exposed Lys groups provide a catalytic substrate for plasminogen activation due to increased binding. Carboxypeptidases inhibit plasmin formation by removing exposed lysine residue from the fibrin clot. PZP (pregnancy zone protein) is an anti plasmin inhibitor, that is also upregulated in the plasma of class III patients (Schematic for **B** was adapted from reference [111]).

PAI-1 mainly functions as a regulator of plasmin-mediated pericellular proteolysis in the vasculature [65]. It is also known to function as an inhibitor of thrombin mediated hemostasis under certain conditions [16,17]. PAI-1 also regulates cell-cell adhesion by inhibiting adhesive actions between α_v_β_3_-integrins and vitronectin. PAI-1 binds to vitronectin (*K_d_* = 50–190 nM) [96], near the RGD cognate binding site for α_v_β_3_ integrin, thus putative adhesive interactions between α_v_β_3_ and vitronectin are sterically hindered [97]. A comprehensive listing of its pleotropic functions can be identified using Gene ontology analysis (http://geneontology.org). We used the program DAVID (Database for Annotation, Visualization, and Integrated Discovery) [98,99] to characterize the functional biology of proteins whose upregulation could be related to the enrichment of PAI-1 in our class III cohort (e.g., TNFα, TNFβ, RhoA activity, ILα, IL6, complement (C3a, C5a, C5b-9)). Based on our data, it is conceivable that class III patients experience significant tissue injury from the very robust increase in proinflammatory cytokines (Figure 6) and complement (Table 4). Vascular damage and exposure of sub endothelial tissue factor (TF) to circulation coagulation factors, triggers coagulation cascade which generates thrombin (*cf.* spot #67) [39,100,101,102,103,104]. Anticoagulant proteins such as tissue plasminogen activator (t-PA), act to produce local plasmin at the endothelial surface to degrade thrombin-generated fibrin clots into soluble degradation products [17,47,105,106]. Partially degraded fibrin contains *C*-terminal lysine residues that catalyze fibrinolysis by providing cognate binding sites for t-PA, plasminogen and plasmin [107] as shown in Figure 10. On this basis, tranexamic acid a synthetic analog of lysine is used as a *C*-terminal lysine decoy to inhibit plasminogen activation in the clinic to control hemorrhage [58,108]. Carboxypeptidases catalyze the removal of the *C*-terminal amino acid residues in peptides and proteins [46,48,49,109,110] and thus inhibit plasmin generation and fibrinolysis. Carboxypeptidase N (spot #193) is an intrinsically active metallocarboxypeptidase [46,48,107] that regulates biologically active peptides including complement anaphylatoxins (C3a, C4a, and C5a) and kinins by removing carboxy-terminal arginine or lysine. CPN has also been shown to exhibit substantial antifibrinolytic activity induced by plasmin [48]. Thrombin activatable fibrinolysis inhibitor (TAFI) is a carboxypeptidase zymogen [107] that shares significant substrate specificity with CPN [48], and could also be involved in prothombotic activity in HCPS. TAFI is converted to its short lived (t_1/2_ = 8–15 min) [48] active form (TAFIa) by thrombin or plasmin whereas, the antifibrinolytic activity of CPN, is conferred by the proteolytic activity of plasmin [48]. Plasma levels of CPN are significantly depleted in class III (Table 3 spot #193) relative to class II, even though the consumption of *anaphylatoxin-generating* complement appears to be comparable throughout the illness spectrum (Table 2 and Table 3 spots #150, 166, 167, 370). We hypothesize that class III patients suffer significant endothelial damage (implied by the significant up-regulation of elongation factor 2 in class III (spot #53 in Table 3)). We suggest that the consumption of CPN was likely caused by anti-fibrinolytic activity, an argument, which aligns with the upregulation of pregnancy zone protein (spot #31) and PAI-1 expression. Conversely depletion of CPN could leave the host less protected against analphylatoxins.

This would suggest that PAI-1 might be a response to the severe pathology as it is expected to reduce plasmin activity and possibly thrombin activity in class III patients, where PAI-1 levels are extremely high [16,17,18].

## 4. Conclusions 

In summary, in this study we have examined the plasma proteome of hantavirus patients in order to determine protein patterns comprising edemagenic factors and biomarkers associated with the hosts’ response to HCPS. The identified factors could then be used as early indicators of the clinical trajectory of disease. The most significant finding of our study is the evidence of severe prothrombotic response in terminal class III patients relative to surviving patients, which is atypical of HPCS caused by SNV [3,9,55,56], but prevalent for hemorrhagic fevers caused by Dengue, Marburg, Ebola, Hantaan and Lassa as well as HFRS causing hantaviruses [101,112,113,114,115]. Because bleeding complications are normally not considered to be a factor in HCPS, this finding is highly significant, as proactive treatment of coagulation complications in severe HCPS patients could be life saving. Thus a prospective study of severe SNV patients would be an interesting examination of physiological conditions that contribute to the overexpression of PAI-1 in class III etiology.

## Supplementary Files

Supplementary File 1
