# Supplementary material for: Elevated Cytokines, Thrombin and PAI-1 in Severe HCPS Patients Due to Sin Nombre Virus
Source: Viruses. 2015 Feb 10;7(2):559–89. doi: 10.3390/v7020559 (PMC4353904; doi:10.3390/v7020559)
Supplement: Supplementary File 1 [file viruses-07-00559-s001.zip › viruses-69843-Supplementary Files/Supplementary Figures.pdf]

Fig. S1

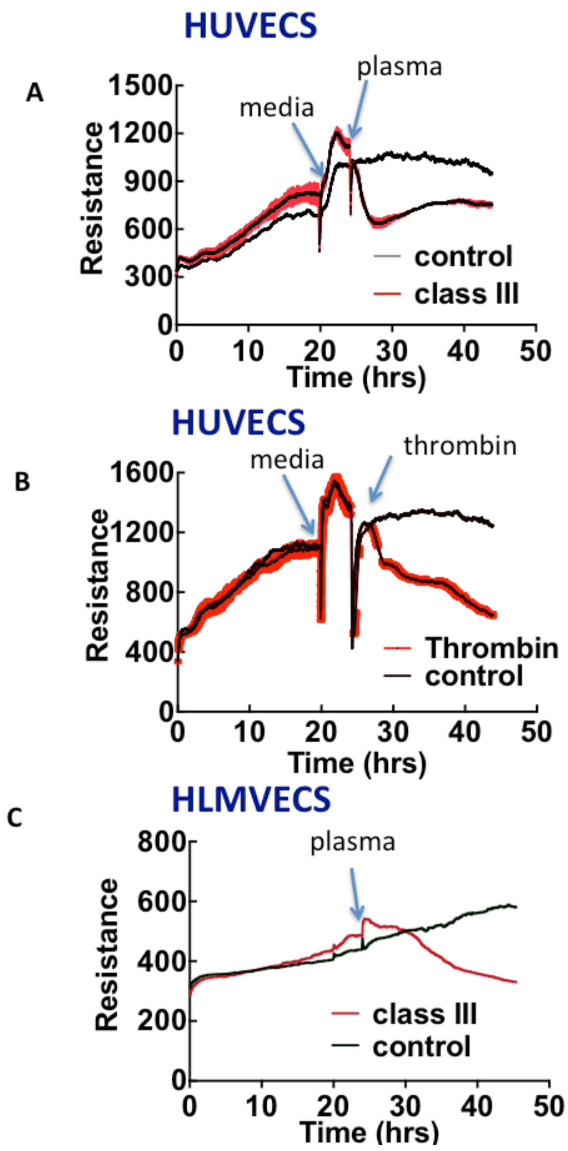

Figure S1. ECIS profile of the effect of a class III HCPS patient plasma on cell barrier properties of endothelial cells: **A**. HUVEC cells were plated in electrode-containing dishes was at 10^5 cells/cm^2 and allowed to attach, spread, and organize for at least 20 hours. Cellular impedance was measured continuously at a single frequency of 4000Hz. Increase in resistance corresponds to increasing cell barrier function. The rapid increase in resistance at about 20 hours occurred after the media was exchanged. Patient and control plasma were added to monolayers. **B.** Effects of thrombin on HUVECs **C**. Effect on plasma on HLMVECs.

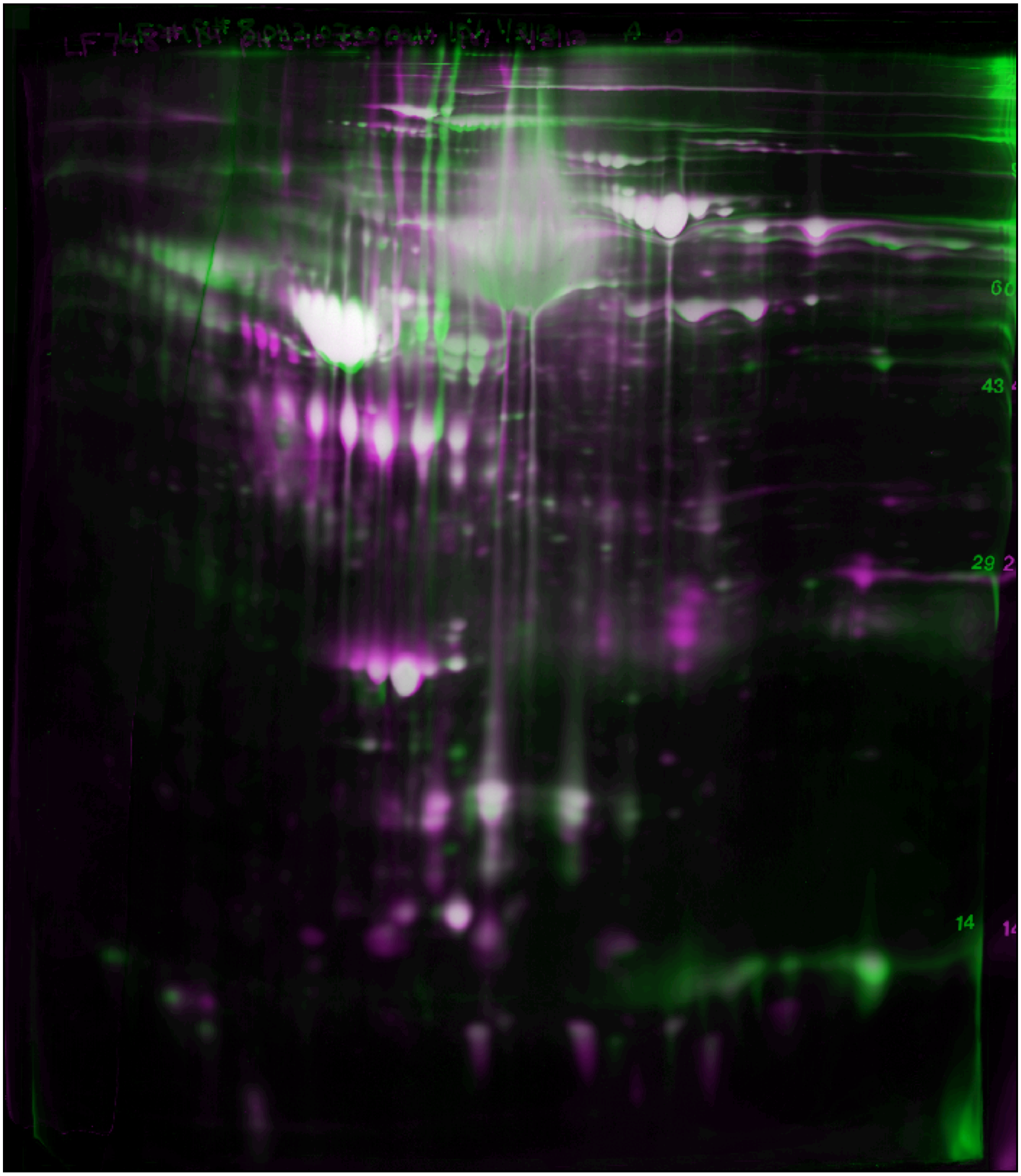

Figure S2A. Overlay image showing pooled class I HCPS plasma in green overlaying Control in magenta. Image appears white where spots of similar intensity overlay each other.

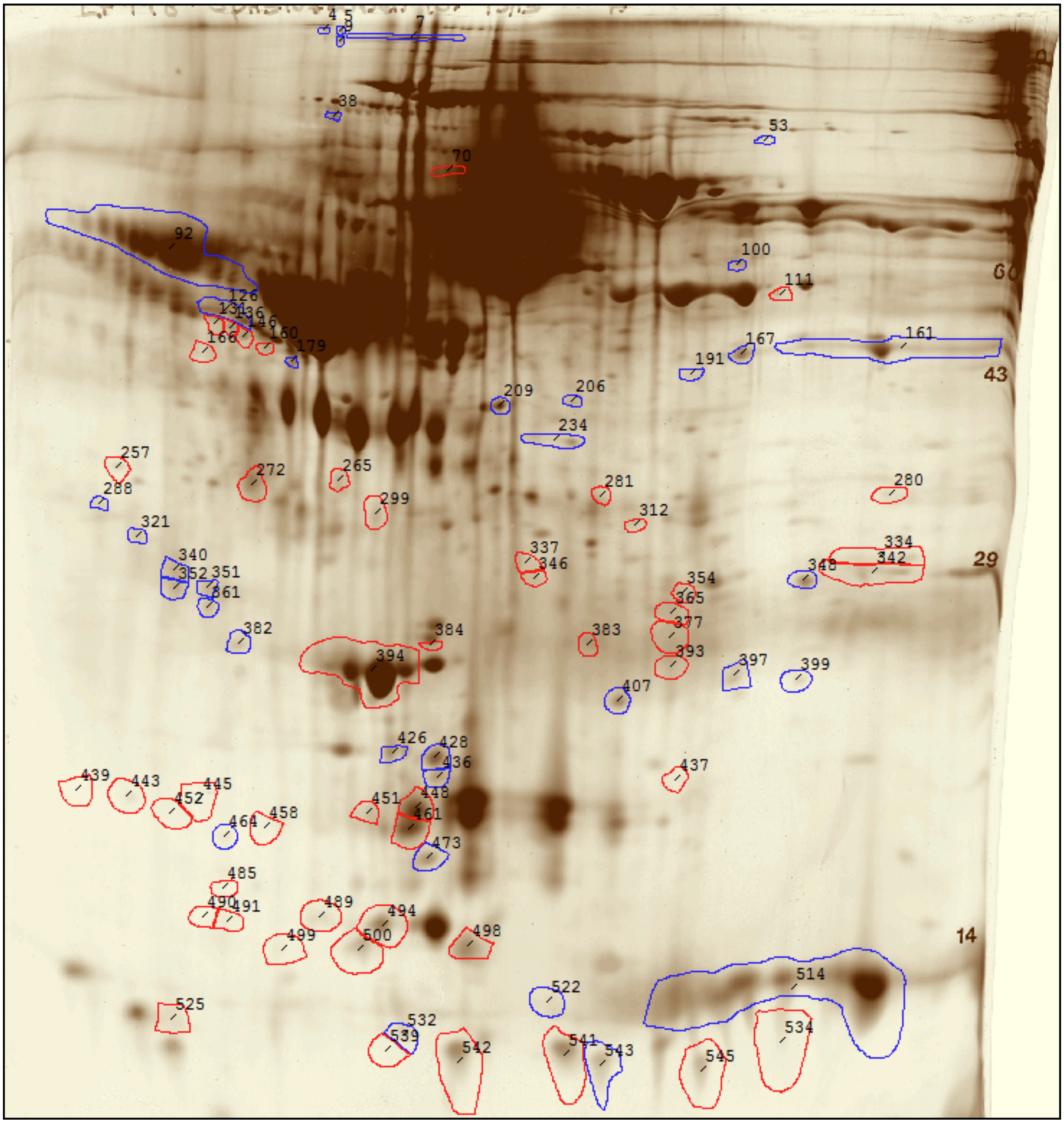

Figure S2B. 2D Gel Difference Image of pooled class I HCPS plasma versus Control. Polypeptide spots *Increased* in I vs control are outlined in Blue, while spots *Decreased* in I vs control are outlined in Red. See Table 1 for spot measurements.

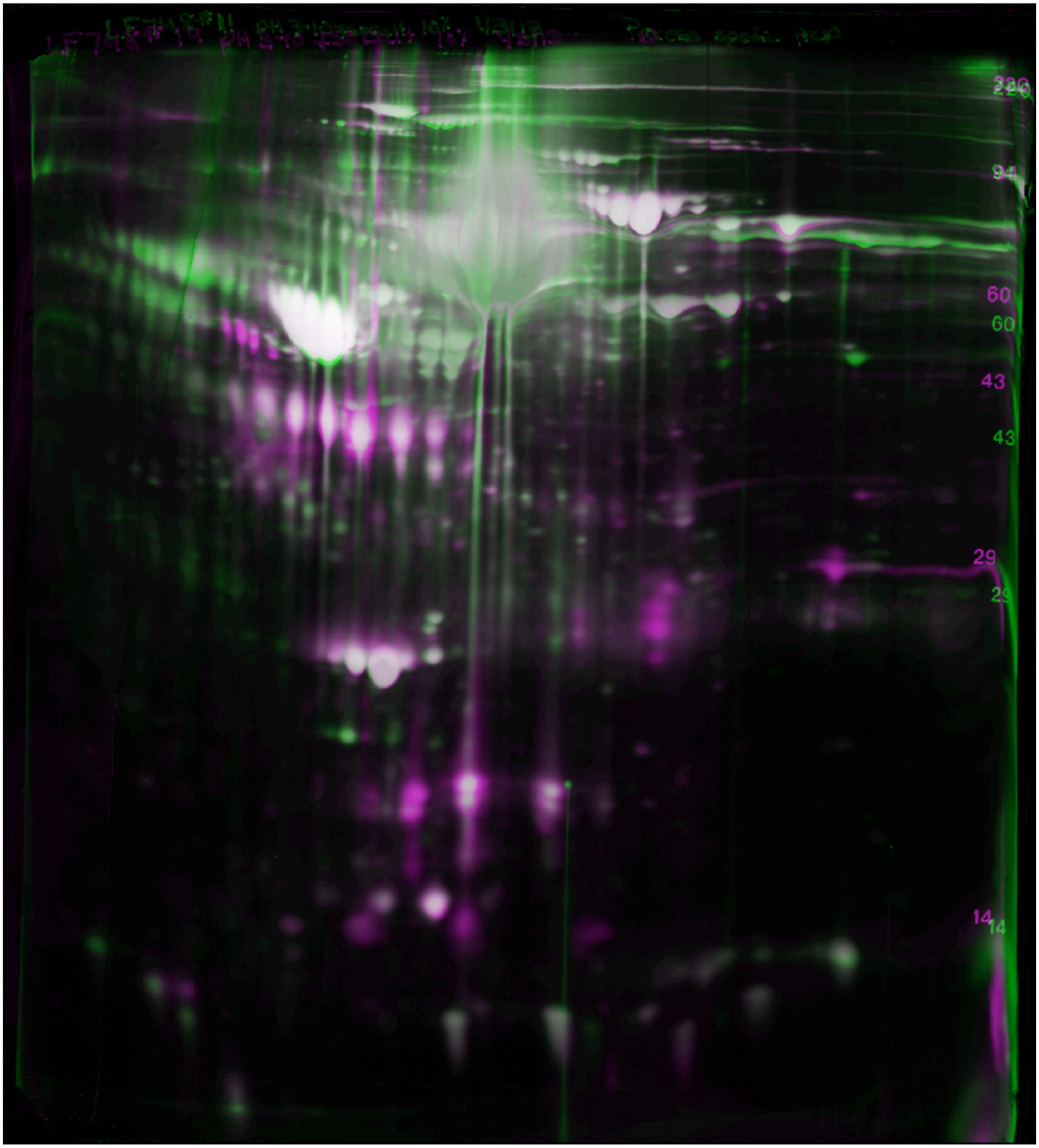

Figure S3A. Overlay image showing classII HCPS plasma in green overlaying sample Control in magenta. Image appears white where spots of similar intensity overlay each other.

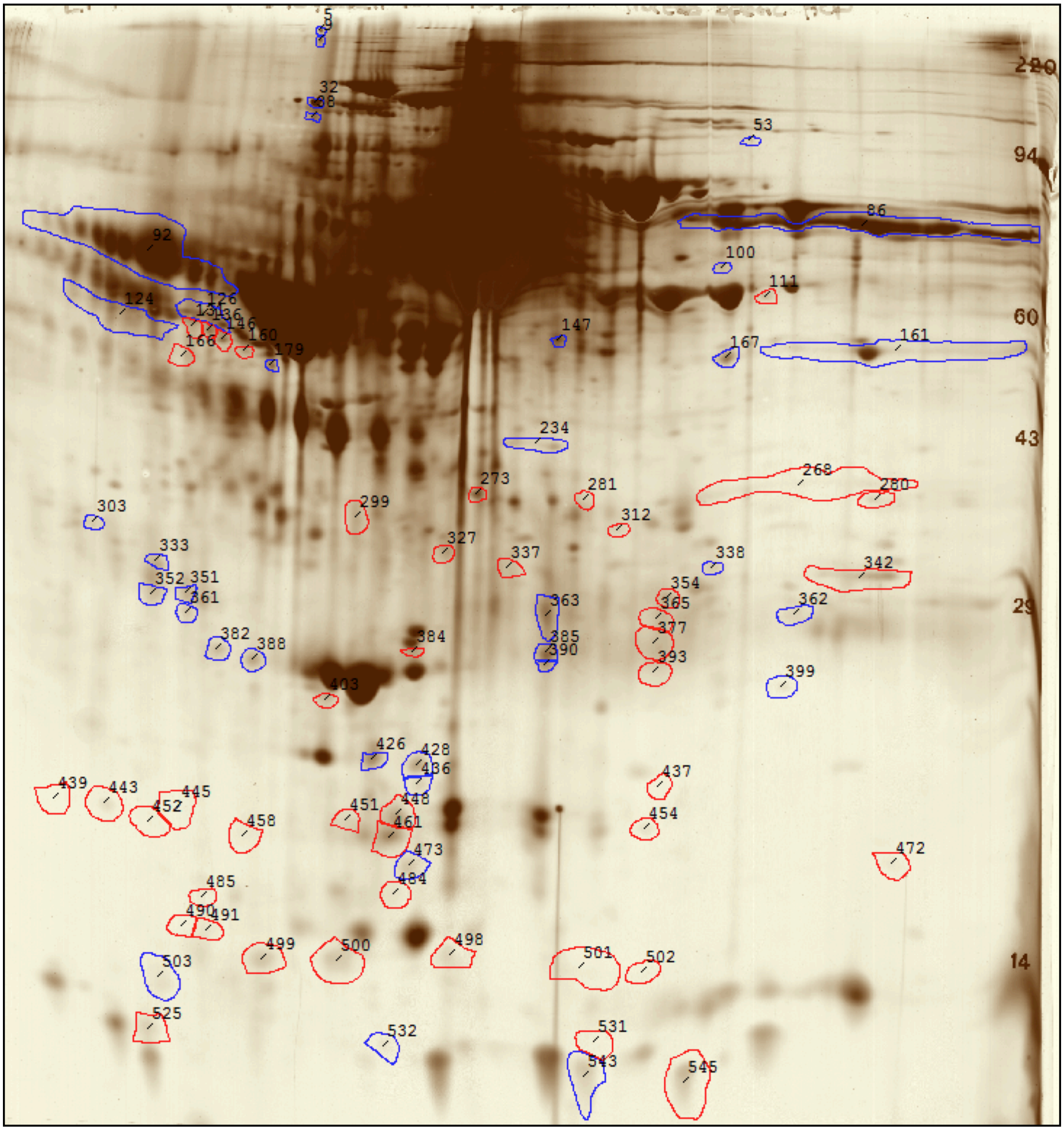

Figure S3B. 2D Gel Difference Image of class II HCPS versus Control gels. Polypeptide spots *Increased* in II vs control are outlined in Blue, while spots *Decreased* in II vs control are outlined in Red. See Table 1 for spot measurements.

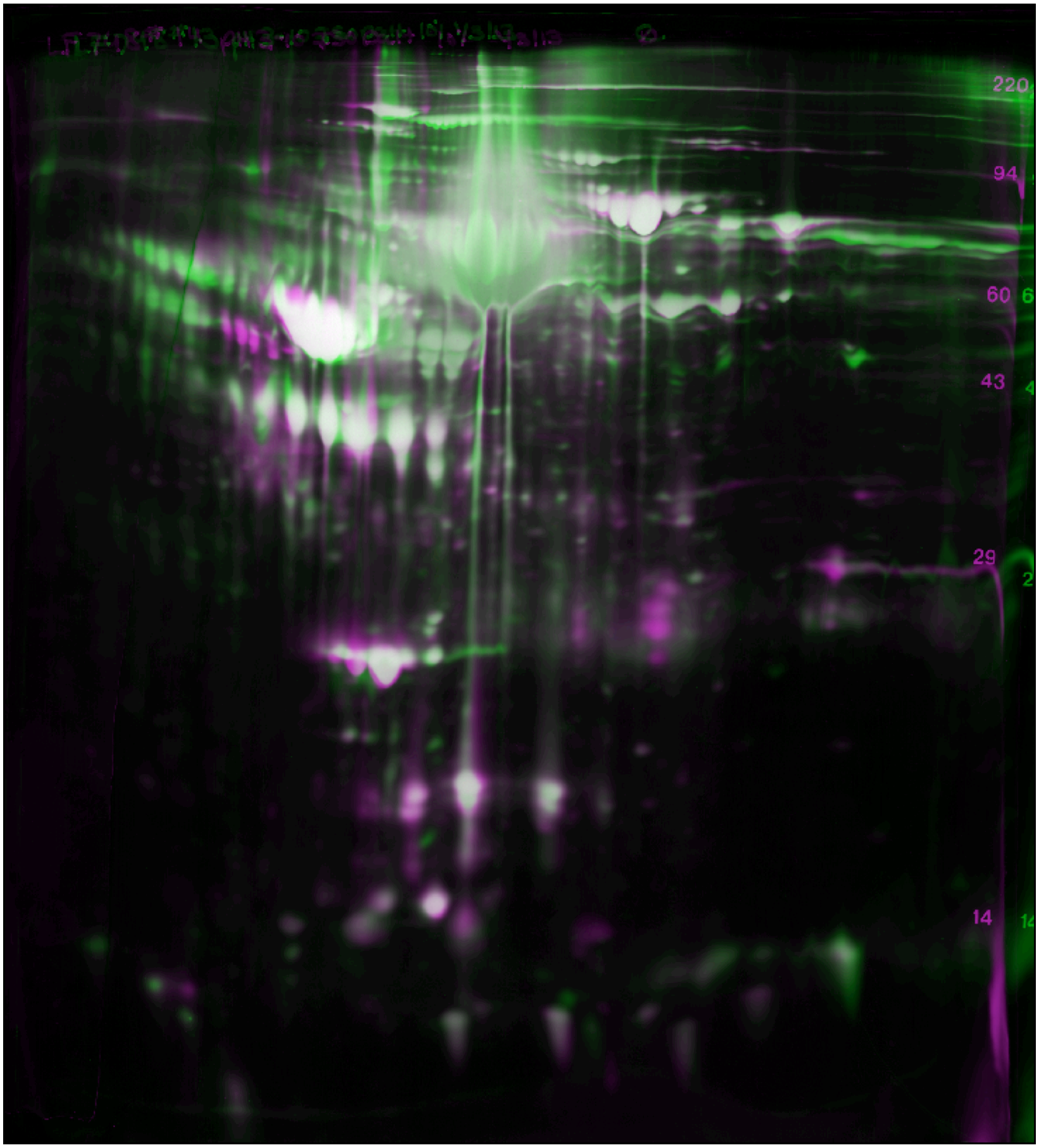

Figure S4A. Overlay image showing class III HCPS in green overlaying Control gel in magenta. Image appears white where spots of similar intensity overlay each other.

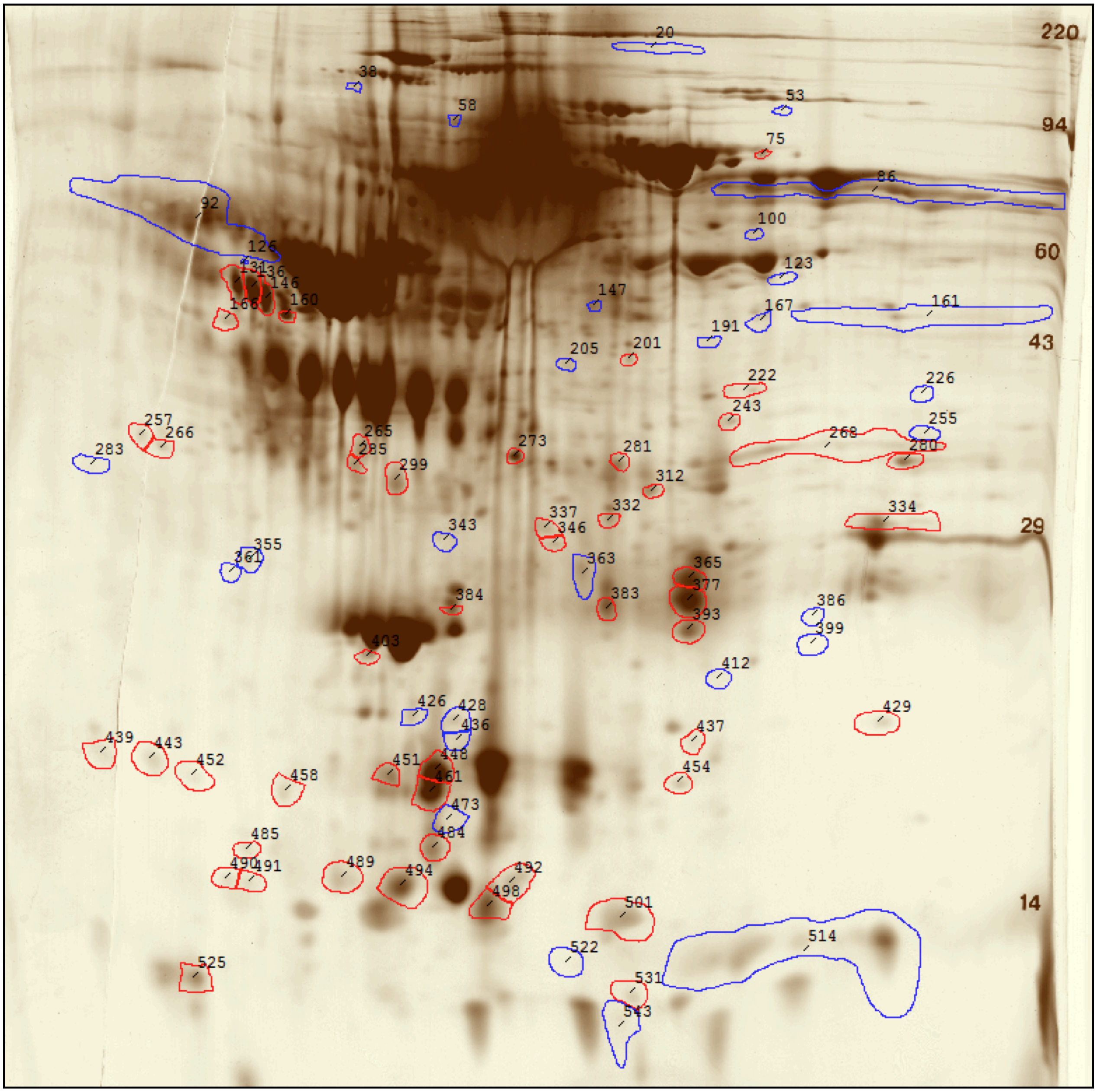

Figure S4B. 2D Gel Difference Image of class III vs Control. Polypeptide spots *increased* in III vs Control are outlined in Blue, while spots *decreased* in III vs control are outlined in Red.

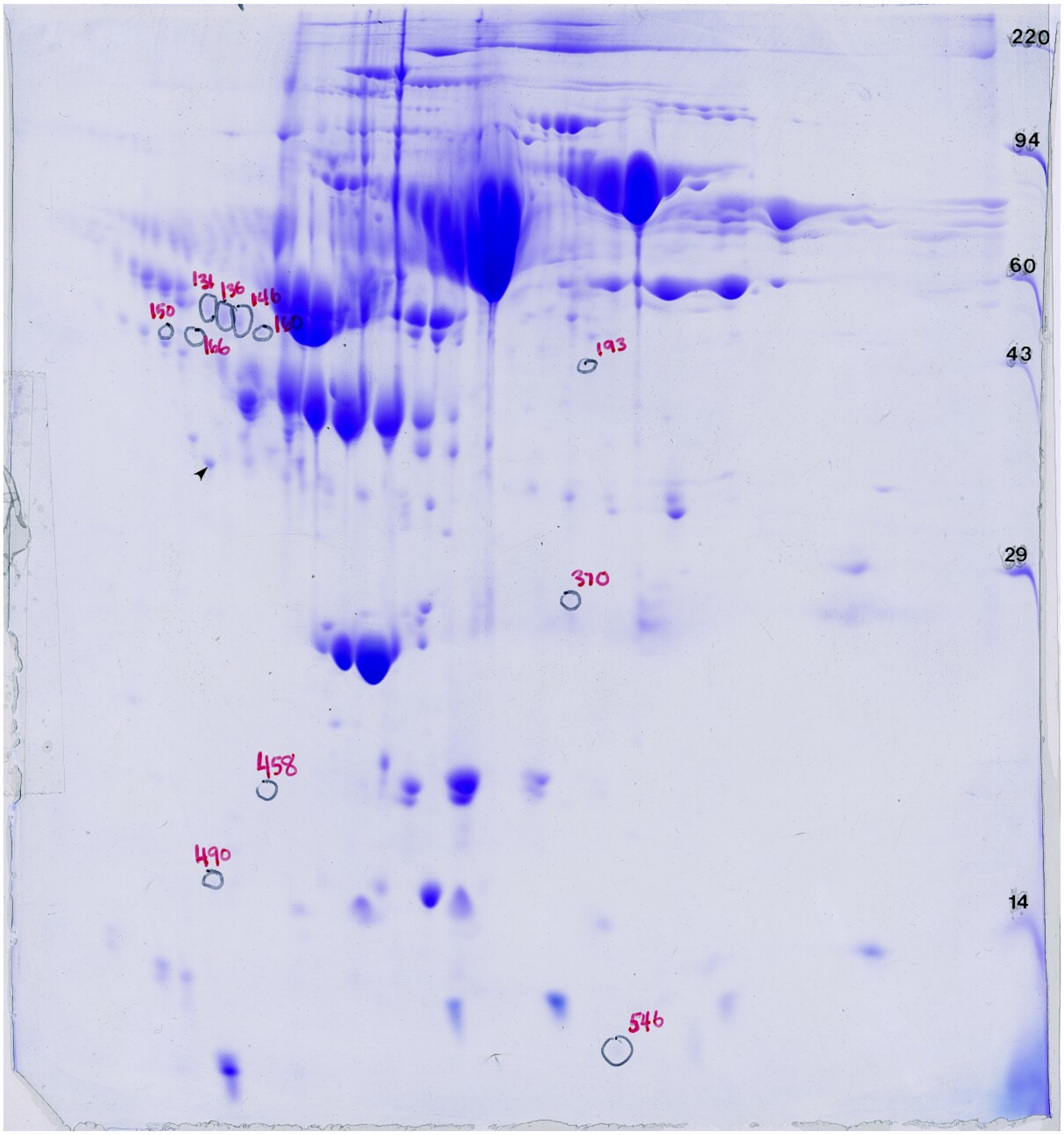

Fig. S5. Coomassie blue-stained 2D gel of control sample showing spots selected for mass spectrometry

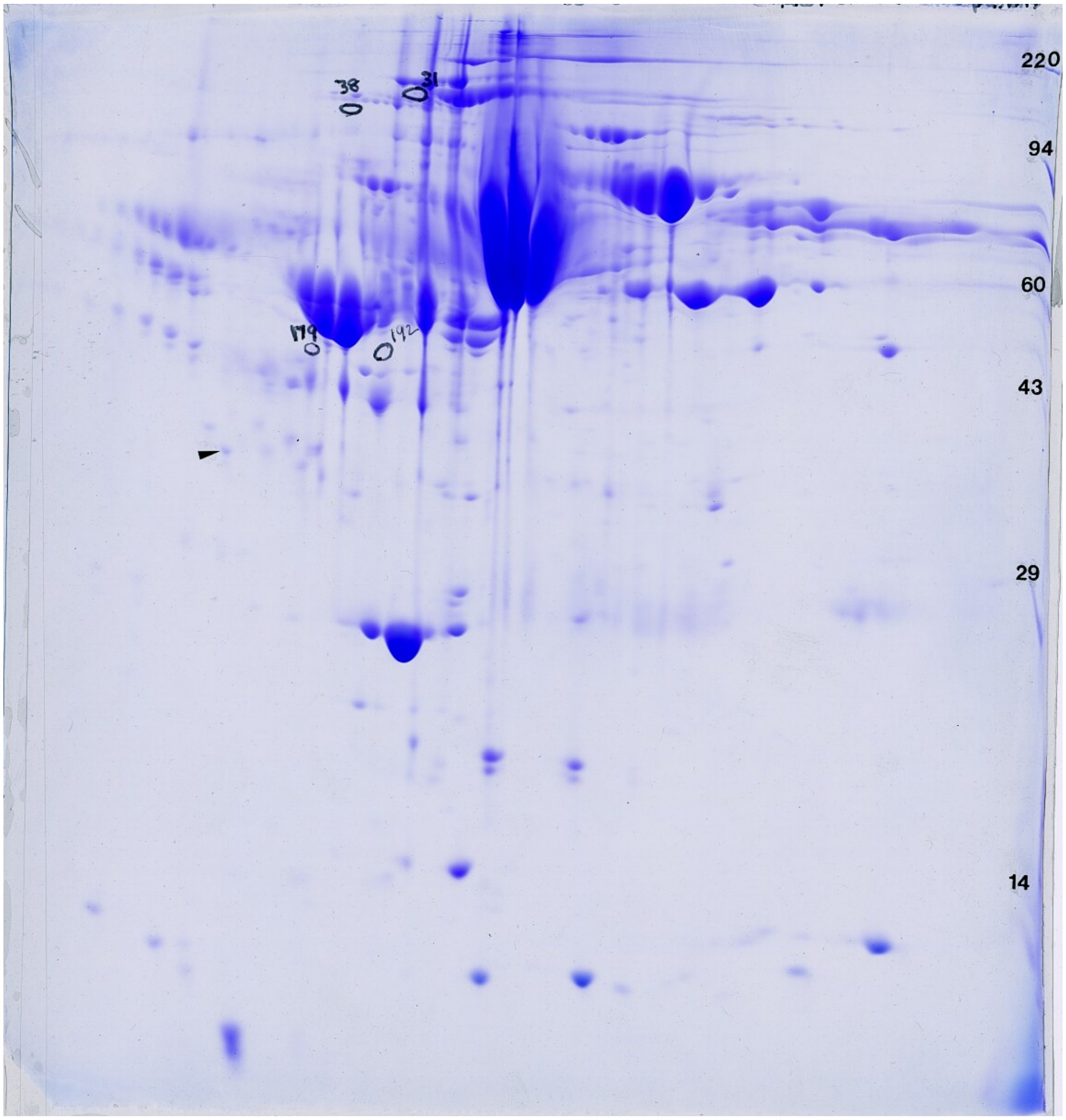

Figure S6. Coomassie blue-stained 2D gel of Class II sample showing spots selected for mass spectrometry

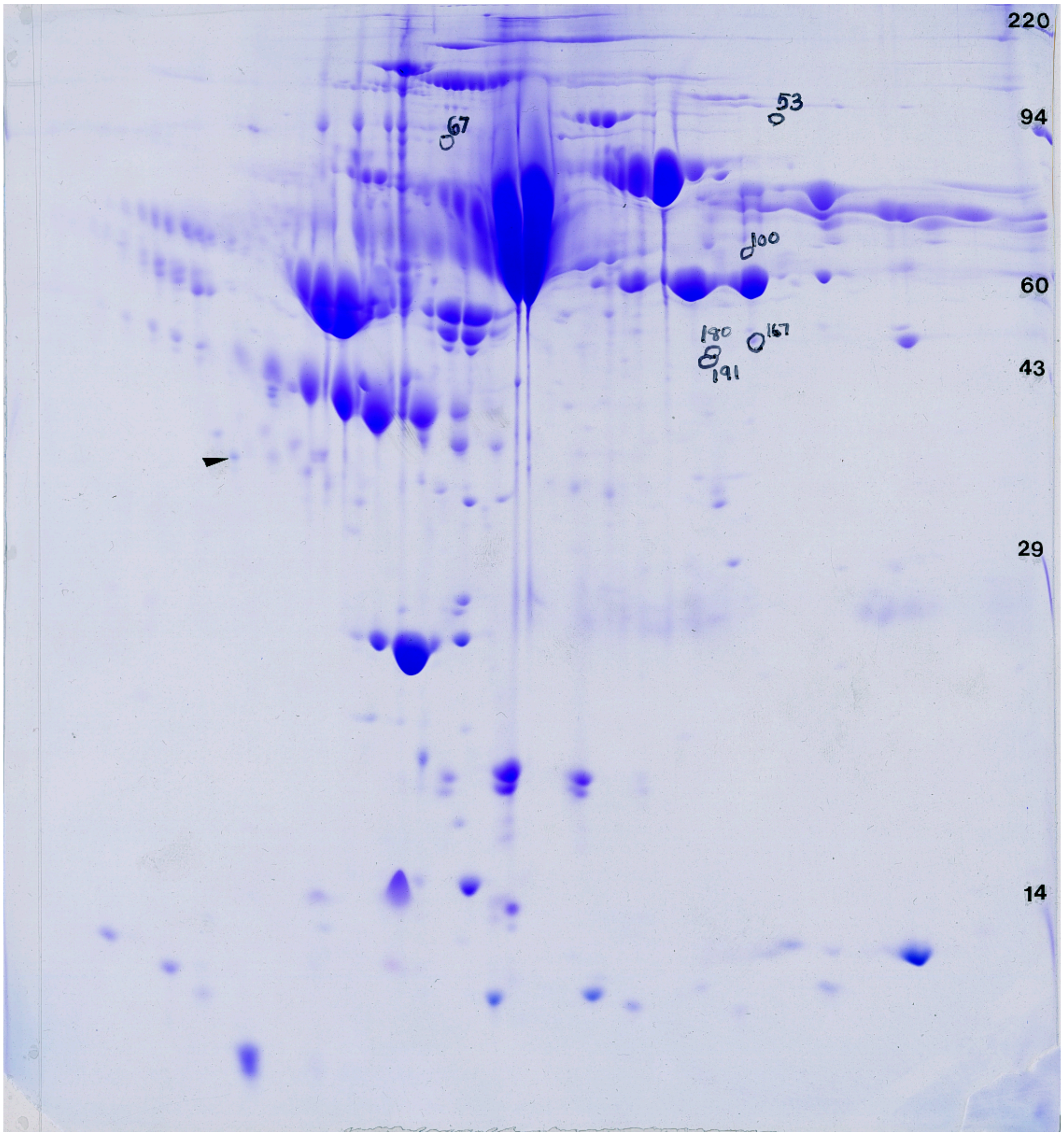

Figure S7. Coomassie blue-stained 2D gel of Class III sample showing spots selected for mass spectrometry
