# Supplementary material for: Elevated Cytokines, Thrombin and PAI-1 in Severe HCPS Patients Due to Sin Nombre Virus
Source: Viruses. 2015 Feb 10;7(2):559–89. doi: 10.3390/v7020559 (PMC4353904; doi:10.3390/v7020559)
Supplement: Supplementary File 1 [file viruses-07-00559-s001.zip › viruses-69843-Supplementary Files/Supplementary Tables.pdf]

Table S1. Tryptic peptides from Spot #31 measured by MALDI-TOF. The protein was identified as pregnancy zone protein, SwissProt accession P20742. Measured masses from the MALDI-TOF spectrum are compared to calculated values. Masses listed represent 21% sequence coverage. MS-Fit MOWSE score: 5.89e+09; Mascot score: 74, expect value: 7.4e-04

Measured mass, amu (average)	Calculated mass, amu (average)	error, amu	Sequence Position Start	End	Sequence
1505.39	1505.74	-0.35	81	92	K.DLFHCVSFTLPR.I
3177.41	3177.67	-0.26	117	144	R.NTVLVLNTQSLVFVQTDKPMYKPGQTVR.F
1583.64	1583.83	-0.18	158	170	R.NELIPLIYLENPR.R
973.29	973.13	0.17	174	181	R.IAQWQSLK.L
1031.16	1031.12	0.04	204	213	R.VVVQTESGGR.I
1683.67	1683.94	-0.27	214	227	R.IQHPFTVEEFVLPK.F
2893.40	2893.13	0.28	280	303	K.QEVCEEFSQQLNSNGCITQQVHTK.M
2153.45	2153.53	-0.07	304	321	K.MLQITNTGFEMKLRVEAR.I + Oxidation (M)
1602.73	1602.70	0.03	322	335	R.IREEGTDLEVTANR.I
1631.54	1631.91	-0.37	357	371	R.QGIPFFAQVLLVDGK.G
1458.53	1458.59	-0.06	504	517	R.SGTHTLPVESGDMK.G
1474.54	1474.59	-0.05	504	517	R.SGTHTLPVESGDMK.G + Oxidation (M)
1862.86	1863.07	-0.21	518	535	K.GSFALSFPVESDVAPIAR.M
2934.26	2934.40	-0.14	595	621	R.AVDQSVLLMKPEAELSVSSVYNLLTVK.D
1317.56	1317.58	-0.02	673	683	K.VFTNSKIRKPK.S
2096.93	2097.31	-0.37	773	793	K.AGAFCLSEDAGLGISSTASLR.A
1501.27	1501.60	-0.33	848	859	K.GEESYCICGNER.Q
1160.00	1160.32	-0.32	860	869	R.QTLSWTVTPK.T
2406.19	2406.52	-0.33	1021	1040	R.QLNYKHQDGSYSTFGERYGR.N
3240.96	3241.36	-0.40	1455	1482	K.VYDYYETDESVVAEYIAPCSTDTEHGNV.-

Table S2. Tryptic peptides from Spot #38 measured by MALDI-TOF. The protein was identified as alpha-1-antitrypsin, SwissProt accession P01009. Measured masses from the MALDI-TOF spectrum are compared to calculated values. Masses listed represent 33% sequence coverage. MS-Fit MOWSE score: 2.12e+05; Mascot score: 82, expect value: 1.2e-04

Measured mass, amu (average)	Calculated mass, amu (average)	error, amu	Sequence Position Start	End	Sequence
1641.76	1641.86	-0.10	50	63	K.ITPNLAEFAFSLYR.Q
2575.06	2574.84	0.23	126	149	R.TLNQPDSQLQLTTGNGLFLSEGLK.L
2058.19	2058.16	0.03	161	178	K.LYHSEAFTVNFGDTEEAK.K
1008.11	1008.08	0.03	180	187	K.QINDYVEK.G
1090.25	1090.23	0.02	218	225	K.WERPFEVK.D
1891.55	1891.94	-0.39	226	241	K.DTEEEDFHVDQVTTVK.V
1092.11	1092.35	-0.24	259	267	K.LSSWVLLMK.Y + Oxidation (M)
1803.90	1804.01	-0.11	284	298	K.LQHLENELTHDIITK.F
1856.12	1856.19	-0.07	390	404	K.FNKPFVFLMIEQNTK.S
1561.51	1561.84	-0.33	405	418	K.SPLFMGKVVNPTQK.- + Oxidation (M)

Table S3. Tryptic peptides from Spot #179 measured by MALDI-TOF. The protein was identified as haptoglobin, SwissProt accession P00738. Measured masses from the MALDI-TOF spectrum are compared to calculated values. Masses listed represent 27% sequence coverage. MS-Fit MOWSE score: 8.15e+02; Mascot score: 45, expect value: 5.8e-01

Measured mass, amu (average)	Calculated mass, amu (average)	error, amu	Sequence Position Start	End	Sequence
2635.08	2635.06	0.02	229	251	K.QLVEIEKVVLHPNYSQVDIGLIK.L
980.18	980.08	0.11	278	286	R.VGYVSGWGR.N
1721.56	1721.99	-0.43	298	311	K.YVMLPVADQDQCIR.H
2186.02	2186.51	-0.49	326	345	K.SPVGVQPILNEHTFCAGMSK.Y
3889.82	3890.07	-0.25	346	379	K.YQEDTCYGDAGSAFAVHDLEEDTWYATGILSFDK.S
1203.44	1203.34	0.10	392	401	K.VTSIQDWVQK.T

Table S4. Tryptic peptides from Spot #67 measured by MALDI-TOF. Three proteins were identified:
(A) Serum albumin, SwissProt accession P02790. Measured masses from the MALDI-TOF spectrum are compared to calculated values. Masses listed represent 28% sequence coverage. MS-Fit MOWSE score: 5.65e+04; Mascot score: 56, expect value: 4.6e-02.

Measured mass, amu (average)	Calculated mass, amu (average)	error, amu	Sequence Position Start	End	Sequence
1149.14	1149.22	-0.07	25	34	R.DAHKSEVAHR.F
1728.69	1728.88	-0.19	118	130	K.QEPERNECFLQHK.D
2664.59	2665.01	-0.41	139	160	R.LVRPEVDVMCTAFHDNEETFLK.K
927.33	927.05	0.28	162	168	K.YLYEIAR.R
2128.81	2129.31	-0.50	265	281	K.VHTECCHGDLLECADDR.A
2989.11	2989.25	-0.13	311	337	K.SHCIAEVENDEMPADLPSLAADFVESK.D
1639.79	1639.87	-0.08	348	360	K.DVFLGMFLYEYAR.R + Oxidation (M)
1467.55	1467.71	-0.16	361	372	R.RHPDYSVVLLLR.L
1311.14	1311.53	-0.39	362	372	R.HPDYSVVLLLR.L
2045.35	2045.33	0.02	397	413	K.VFDEFKPLVEEPQNLIK.Q
960.26	960.13	0.13	427	434	K.FQNALLVR.Y
1213.10	1213.43	-0.33	461	469	K.CCKHPEAKR.M
1924.80	1925.17	-0.36	509	524	R.RPCFSALEVDETYVPK.E

(B) Prothrombin, SwissProt accession P00734. Measured masses from the MALDI-TOF spectrum are compared to calculated values. Masses listed represent 18% sequence coverage. MS-Fit MOWSE score: 6.13e+03; Mascot score: 40, expect value: 1.8

Measured mass, amu (average)	Calculated mass, amu (average)	error, amu	Sequence Position Start	End	Sequence
2461.14	2460.81	0.33	178	198	R.RQECSIPVCGQDQVTVAMTPR.S
1977.75	1978.15	-0.40	248	263	K.HQDFNSAVQLVENFCR.N
1561.60	1561.65	-0.05	315	327	R.TATSEYQTFFNPR.T
2026.77	2026.27	0.50	328	345	R.TFGSGEADCGLRPLFEKK.S
1203.97	1204.44	-0.46	437	446	R.NIEKISMLEK.I

1220.49	1220.44	0.06	437	446	R.NIEKISMLEK.I + Oxidation (M)
1515.54	1515.82	-0.28	441	452	K.ISMLEKIYIHPR.Y + Oxidation (M)
2246.24	2246.67	-0.43	518	537	K.GQPSVLQVVNLPIVERPVCK.D
1189.23	1189.32	-0.09	600	608	K.YGFYTHVFR.L

(C) Hemopexin, SwissProt accession P02790. Measured masses from the MALDI-TOF spectrum are compared to calculated values.Masses listed represent 23% sequence coverage. MS-Fit MOWSE score: 4.53e+03

Measured mass, amu (average)	Calculated mass, amu (average)	error, amu	Sequence Position Start	End	Sequence
1220.50	1220.37	0.13	92	102	K.NFPSPVDAAFR.Q
2378.63	2378.71	-0.08	131	151	K.LLQDEFPGIPSPLDAAVECHR.G
1509.82	1509.72	0.10	198	208	R.YYCFQGNQFLR.F
1156.28	1156.36	-0.08	226	234	R.DYFMPCPGR.G
2512.56	2512.85	-0.29	348	371	K.EVGTPHGIILDSVDAAFICPGSSR.L
1924.81	1924.37	0.44	372	386	R.LHIMAGRRLWWLDLK.S + Oxidation (M)
1750.09	1750.03	0.06	447	462	K.ALPQPQNVTSLLGCTH -

Table S5. Tryptic peptides from Spot #100 measured by MALDI-TOF. The protein was identified as 6-phosphofructo-2-kinase/fructose-2,6-bisphosphatase 4, SwissProt accession Q16877. Measured masses from the MALDI-TOF spectrum are compared to calculated values. Masses listed represent 32% sequence coverage. MS-Fit MOWSE score: 4.68e+04; Mascot score: 55, expect value: 6.1e-02

Measured mass, amu (average)	Calculated mass, amu (average)	error, amu	Sequence Position Start	End	Sequence
1481.69	1481.70	0.00	2	14	M.ASPRELTQNPLKK.I
2071.15	2071.39	-0.24	15	31	K.IWMPYSNGRPALHACQR.G
1168.53	1168.26	0.27	72	80	R.EFNVGQYRR.D
1671.72	1671.80	-0.08	88	101	K.SFEFFLPDNEEGLK.I
2336.90	2336.47	0.43	117	137	R.FLSEEGGHVAVFDATNTTRER.R
1619.31	1618.94	0.37	238	250	R.IVYYLMNIHVTPR.S
1239.01	1239.34	-0.33	257	267	R.HGESELNLKGR.I
1711.08	1711.02	0.06	383	396	R.QENVLVICHQAVMR.C
2170.95	2170.57	0.38	397	414	R.CLLAYFLDKAAEQLPYLK.C
1032.32	1032.19	0.13	406	414	K.AAEQLPYLK.C
2673.80	2674.08	-0.28	423	446	K.LTPVAYGCKVESIFLNVAAVNTHR.D

Table s6. Tryptic peptides from Spot #167 measured by MALDI-TOF. The protein was identified as a fragment of complement C3, NCBI accession gi:194384410. Measured masses from the MALDI-TOF spectrum are compared to calculated values. Masses listed represent 7% sequence coverage. MS-Fit MOWSE score: 1.01e+05; Mascot score: 49, expect value: 2.5e-01

Measured mass, amu (average)	Calculated mass, amu (average)	error, amu	Sequence Position Start	End	Sequence
954.93	955.09	-0.16	693	699	K.CCEDGMR.E
1038.25	1038.22	0.03	713	721	R.FISLGEACK.K
1859.18	1859.17	0.01	722	735	K.KVFLDCCNYITELR.R
1730.91	1731.00	-0.09	723	735	K.VFLDCCNYITELR.R
1816.71	1816.92	-0.20	749	764	R.SNLDEDIIAEENIVSR.S
2070.23	2070.41	-0.18	862	879	K.VRVELLHNPAFCSLATTK.R
1471.62	1471.62	0.00	914	926	K.AAVYHHFISDGVR.K
2755.90	2755.85	0.05	955	979	R.EGVQKEDIPPADLSDQVPDTESETR.I
2214.24	2214.25	-0.01	960	979	K.EDIPPADLSDQVPDTESETR.I
2157.30	2157.40	-0.10	980	999	R.ILLQGTPVAQMTEDAVDAER.L

Table S7. Tryptic peptides from Spot #180 measured by MALDI-TOF. The protein was identified as plasminogen activator inhibitor 1, SwissProt accession P05121. Measured masses from the MALDI-TOF spectrum are compared to calculated values. Masses listed represent 36% sequence coverage. MS-Fit MOWSE score: 1.02e+07; Mascot score: 110, expect value: 2e-07

Measured mass, amu (average)	Calculated mass, amu (average)	error, amu	Sequence Position Start	End	Sequence
2450.33	2450.72	-0.39	104	124	K.ELMGPWNKDEISTTDAIFVQR.D
2466.48	2466.72	-0.24	104	124	K.ELMGPWNKDEISTTDAIFVQR.D + Oxidation (M)
1494.58	1494.60	-0.02	112	124	K.DEISTTDAIFVQR.D
1378.66	1378.64	0.02	128	138	K.LVQGFMPHFFR.L
1394.67	1394.64	0.03	128	138	K.LVQGFMPHFFR.L + Oxidation (M)
1795.10	1795.16	-0.06	128	141	K.LVQGFMPHFFRLFR.S
1108.22	1108.16	0.06	146	154	K.QVDFSEVER.A
1664.86	1664.94	-0.08	186	199	R.LVLVNALYFNGQWK.T
1144.16	1144.19	-0.03	200	209	K.TPFPDSSTHR.R
1208.41	1208.32	0.09	301	310	K.FSLETEVDLR.K
1551.73	1551.83	-0.10	311	323	R.KPLENLGMTDMFR.Q
1567.67	1567.83	-0.16	311	323	R.KPLENLGMTDMFR.Q + Oxidation (M)
2601.71	2601.82	-0.11	324	346	R.QFQADFTSLSDQEPLHVAQALQK.V
2304.17	2304.55	-0.38	347	369	K.VKIEVNESGTVASSSTAVIVSAR.M

Table S8. Tryptic peptides from Spot #191 measured by MALDI-TOF. The protein was identified as isocitrate dehydrogenase [NADP] cytoplasmic, SwissProt accession O75874. Measured masses from the MALDI-TOF spectrum are compared to calculated values. Masses listed represent 52% sequence coverage. MS-Fit MOWSE score: 1.38e+08; Mascot score: 157, expect value: 4e-12

Measured mass, amu (average)	Calculated mass, amu (average)	error, amu	Sequence Position Start	End	Sequence
1695.83	1695.87	-0.04	5	20	K.ISGGSVVEMQGDEMTR.I
976.26	976.13	0.13	101	109	R.NILGGTVFR.E
1437.81	1437.77	0.04	120	132	R.LVSGWVKPIIIGR.H
1009.13	1009.03	0.10	133	140	R.HAYGDQYR.A
2507.42	2507.75	-0.32	141	164	R.ATDFVVPGPGKVEITYTPSDGTQK.V
2559.60	2559.83	-0.22	165	187	K.VTYLVHNFEEGGGVAMGMYNQDK.S
1445.37	1445.61	-0.24	223	233	R.FKDIFQEIYDK.Q
903.02	903.00	0.03	244	249	K.IWYEHR.L
1168.17	1168.32	-0.15	261	270	K.SEGGFIWACK.N
3265.27	3265.56	-0.29	271	301	K.NYDGDVQSDSVAQGYGSLGMMTSVLVCPDGK.T
1878.90	1879.03	-0.13	322	338	K.GQETSTNPIASIFAWTR.G
2661.32	2660.99	0.34	351	374	K.ELAFFANALEEVSIETIEAGFMTK.D
1509.28	1509.64	-0.35	389	400	R.SDYLNTFEFMDK.L
2406.18	2405.72	0.46	389	408	R.SDYLNTFEFMDKLGENLKIK.L
914.10	914.10	0.00	401	408	K.LGENLKIK.L

Table S9. Tryptic peptides from Spot #192 measured by MALDI-TOF. The protein was identified as haptoglobin, SwissProt accession P00738. Measured masses from the MALDI-TOF spectrum are compared to calculated values. Masses listed represent 32% sequence coverage. MS-Fit MOWSE score: 3.23e+04; Mascot score: 78, expect value: 3e-4

Measured mass, amu (average)	Calculated mass, amu (average)	error, amu	Sequence Position Start	End	Sequence
920.13	920.02	0.11	171	178	K.GSFPWQAK.M
2634.96	2635.06	-0.10	229	251	K.QLVEIEKVVLHPNYSQVDIGLIK.L
980.18	980.08	0.11	278	286	R.VGYVSGWGR.N
1721.83	1721.99	-0.16	298	311	K.YVMLPVADQDQCIR.H
2186.03	2186.51	-0.48	326	345	K.SPVGVQPILNEHTFCAGMSK.Y
3890.13	3890.07	0.06	346	379	K.YQEDTCYGDAGSAFAVHDLEEDTWYATGILSFDK.S
1359.11	1359.55	-0.43	380	391	K.SCAVAEYGVYVK.V
1203.20	1203.34	-0.14	392	401	K.VTSIQDWVQK.T

Table S10. Tryptic peptides from Spot #131 measured by MALDI-TOF. The protein was identified as alpha-1-antitrypsin,

SwissProt accession P01009. Measured masses from the MALDI-TOF spectrum are compared to calculated values. Masses listed represent 20% sequence coverage. MS-Fit MOWSE score: 2.63e+04; Mascot score: 88, expect value: 2.9e-05.

Measured mass, amu (average)	Calculated mass, amu (average)	error, amu	Sequence Position Start	End	Sequence
1641.76	1641.86	-0.10	50	63	K.ITPNLAEFAFSLYR.Q
2058.22	2058.16	0.06	161	178	K.LYHSEAFTVNFGDTEEAK.K
2186.13	2186.33	-0.20	161	179	K.LYHSEAFTVNFGDTEEAKK.Q
2090.24	2090.38	-0.13	199	215	K.ELDRDTVFALVNYIFFK.G
1275.55	1275.46	0.10	216	225	K.GKWERPFEVK.D
1090.20	1090.23	-0.03	218	225	K.WERPFEVK.D
1417.78	1417.70	0.08	247	257	K.RLGMFNIQHCK.K
1261.36	1261.52	-0.15	248	257	R.LGMFNIQHCK.K
1803.97	1804.01	-0.04	284	298	K.LQHLENELTHDIITK.F

Table S11. Tryptic peptides from Spot #136 measured by MALDI-TOF. The protein was identified as alpha-1-antitrypsin, SwissProt accession P01009. Measured masses from the MALDI-TOF spectrum are compared to calculated values. Masses listed represent 28% sequence coverage. MS-Fit MOWSE score: 7.74e+07; Mascot score: 129, expect value: 2.5e-09.

Measured mass, amu (average)	Calculated mass, amu (average)	error, amu	Sequence Position Start	End	Sequence
1779.85	1779.78	0.08	35	49	K.TDTSHHDQDHPTFNK.I
1641.90	1641.86	0.04	50	63	K.ITPNLAEFAFSLYR.Q
2058.34	2058.16	0.18	161	178	K.LYHSEAFTVNFGDTEEAK.K
2186.34	2186.33	0.01	161	179	K.LYHSEAFTVNFGDTEEAKK.Q
1275.85	1275.46	0.40	216	225	K.GKWERPFEVK.D
1090.32	1090.23	0.09	218	225	K.WERPFEVK.D
1892.08	1891.94	0.14	226	241	K.DTEEEDFHVDQVTTVK.V
1417.73	1417.70	0.03	247	257	K.RLGMFNIQHCK.K
1261.52	1261.52	0.01	248	257	R.LGMFNIQHCK.K
1804.10	1804.01	0.09	284	298	K.LQHLENELTHDIITK.F
921.98	921.95	0.03	299	305	K.FLENEDR.R
1077.95	1078.14	-0.18	299	306	K.FLENEDRR.S
1015.19	1015.20	-0.01	325	334	K.SVLGQLGITK.V

Table S12. Tryptic peptides from Spot #146 measured by MALDI-TOF. The protein was identified as alpha-1-antitrypsin, SwissProt accession P01009. Measured masses from the MALDI-TOF spectrum are compared to calculated values. Masses listed represent 31% sequence coverage. MS-Fit MOWSE score: 8.16e+07; Mascot score: 118, expect value: 3.2e-08.

Measured mass, amu (average)	Calculated mass, amu (average)	error, amu	Sequence Position Start	End	Sequence
1779.92	1779.78	0.15	35	49	K.TDTSHHDQDHPTFNK.I
1641.90	1641.86	0.04	50	63	K.ITPNLAEFAFSLYR.Q
2058.30	2058.16	0.14	161	178	K.LYHSEAFTVNFGDTEEAK.K
2186.22	2186.33	-0.11	161	179	K.LYHSEAFTVNFGDTEEAKK.Q
2090.34	2090.38	-0.03	199	215	K.ELDRDTVFALVNYIFFK.G
1275.89	1275.46	0.44	216	225	K.GKWERPFEVK.D
1090.30	1090.23	0.07	218	225	K.WERPFEVK.D
1892.09	1891.94	0.15	226	241	K.DTEEEDFHVDQVTTVK.V
1261.50	1261.52	-0.01	248	257	R.LGMFNIQHCK.K
1804.08	1804.01	0.07	284	298	K.LQHLENELTHDIITK.F
922.01	921.95	0.06	299	305	K.FLENEDR.R
1015.27	1015.20	0.07	325	334	K.SVLGQLGITK.V

Table S13. Tryptic peptides from Spot #150 measured by MALDI-TOF. The protein was identified as complement C4-B, NCBI accession gi:187771. Measured masses from the MALDI-TOF spectrum are compared to calculated values. Masses listed represent 11% sequence coverage. MS-Fit MOWSE score: 9.18e+04; Mascot score: 51, expect value: 0.17.

Measured mass, amu (average)	Calculated mass, amu (average)	error, amu	Sequence Position Start	End	Sequence
1179.42	1179.33	0.09	701	709	K.RCCQDGVTR.L
2037.12	2037.44	-0.31	829	844	R.VFREFHLHLRLPMSVR.R
2551.85	2551.74	0.11	957	979	R.TLEIPGNSDPNMIPDGDFNSYVR.V
2483.67	2483.73	-0.05	980	1005	R.VTASDPLDTLGSEGALSPGGVASLLR.L
1935.77	1936.12	-0.35	1027	1042	R.YLDKTEQWSTLPPETK.D
2960.39	2960.28	0.12	1031	1055	K.TEQWSTLPPETKDHAVDLIQKGYMR.I + Oxidation (M)
1324.40	1324.44	-0.04	1061	1072	R.KADGSYAAWLSR.G
1541.97	1541.70	0.27	1085	1099	K.VLSLAQEQVGGSPEK.L
2084.91	2085.40	-0.49	1183	1204	K.ASAGLLGAHAAAITAYALTLTK.A
1433.47	1433.59	-0.12	1279	1291	K.AEMADQAAAWLTR.Q
2778.96	2778.98	-0.01	1301	1325	R.STQDTVIALDALSAYWIASHTTEER.G
1367.47	1367.61	-0.14	1380	1390	K.VLRTYNVLDMK.N + Oxidation (M)
983.19	983.14	0.05	1383	1390	R.TYNVLDMK.N

Table S14. Tryptic peptides from Spot #160 measured by MALDI-TOF. The protein was identified as alpha-1-antitrypsin, SwissProt accession P01009. Measured masses from the MALDI-TOF spectrum are compared to calculated values. Masses listed represent 33% sequence coverage. MS-Fit MOWSE score: 4.66e+07; Mascot score: 129, expect value: 2.5e-09.

Measured mass, amu (average)	Calculated mass, amu (average)	error, amu	Sequence Position Start	End	Sequence
1779.84	1779.78	0.07	35	49	K.TDTSHHDQDHPTFNK.I
1642.08	1641.86	0.22	50	63	K.ITPNLAEFAFSLYR.Q
2058.26	2058.16	0.10	161	178	K.LYHSEAFTVNFGDTEEAK.K
2186.04	2186.33	-0.29	161	179	K.LYHSEAFTVNFGDTEEAKK.Q
1090.35	1090.23	0.12	218	225	K.WERPFEVK.D
1892.09	1891.94	0.15	226	241	K.DTEEEDFHVDQVTTVK.V
1261.82	1261.52	0.31	248	257	R.LGMFNIQHCK.K
1804.07	1804.01	0.06	284	298	K.LQHLENELTHDIITK.F
922.03	921.95	0.08	299	305	K.FLENEDR.R
1110.37	1110.26	0.12	315	324	K.LSITGTYDLK.S
1015.17	1015.20	-0.03	325	334	K.SVLGQLGITK.V
1834.07	1833.99	0.08	335	352	K.VFSNGADLSGVTEEAPLK.L

Table S15. Tryptic peptides from Spot #166 measured by MALDI-TOF. The protein was identified as complement C4-B, NCBI accession gi:187771. Measured masses from the MALDI-TOF spectrum are compared to calculated values. Masses listed represent 9% sequence coverage. MS-Fit MOWSE score: 8.53e+06; Mascot score: 49, expect value: 0.27.

Measured mass, amu (average)	Calculated mass, amu (average)	error, amu	Sequence Position Start	End	Sequence
1334.43	1334.54	-0.11	513	523	R.GQIVFMNREPK.R + Oxidation (M)
2551.81	2551.74	0.07	957	979	R.TLEIPGNSDPNMIPDGDFNSYVR.V
2483.63	2483.73	-0.09	980	1005	R.VTASDPLDTLGSEGALSPGGVASLLR.L
1953.26	1953.24	0.02	1009	1026	R.GCGEQTMIYLAPTLAASR.Y
1936.04	1936.12	-0.08	1027	1042	R.YLDKTEQWSTLPPETK.D
1324.21	1324.44	-0.23	1061	1072	R.KADGSYAAWLSR.G
1195.99	1196.27	-0.28	1062	1072	K.ADGSYAAWLSR.G
1541.63	1541.70	-0.07	1085	1099	K.VLSLAQEQVGGSPEK.L
1433.87	1433.59	0.28	1279	1291	K.AEMADQAAAWLTR.Q
983.08	983.04	0.04	1292	1300	R.QGSFQGGFR.S
2778.94	2778.98	-0.03	1301	1325	R.STQDTVIALDALSAYWIASHTTEER.G

Table S16. Tryptic peptides from Spot #193 measured by MALDI-TOF. The protein was identified as carboxypeptidase N catalytic chain, SwissProt accession P15169. Measured masses from the MALDI-TOF spectrum are compared to calculated values. Masses listed represent 37% sequence coverage. MS-Fit MOWSE score: 3.47e+07; Mascot score: 121, expect value: 1.6e-08.

Measured mass, amu (average)	Calculated mass, amu (average)	error, amu	Sequence Position Start	End	Sequence
1187.32	1187.33	0.00	38	47	K.VQNECPGITR.V
2586.04	2585.86	0.18	59	80	R.HLYVLEFSDHPGIHEPLEPEVK.Y
1417.59	1417.55	0.04	81	93	K.YVGNMHGNEALGR.E
1433.58	1433.55	0.03	81	93	K.YVGNMHGNEALGR.E + Oxidation (M)
1958.23	1958.26	-0.03	94	108	R.ELMLQLSEFLCEEFR.N
1974.37	1974.26	0.11	94	108	R.ELMLQLSEFLCEEFR.N + Oxidation (M)
1085.37	1085.25	0.12	114	122	R.IVQLIQDTR.I
3065.58	3065.46	0.12	123	151	R.IHILPSMNPDGYEVAAAQGPNKPGYLVGR.N
3081.78	3081.46	0.32	123	151	R.IHILPSMNPDGYEVAAAQGPNKPGYLVGR.N + Oxidation (M)
1745.03	1744.91	0.13	176	190	K.YGGPNHHLPLPDNWK.S
1752.93	1753.01	-0.07	329	343	R.EALIQFLEQVHQGIK.G
3795.46	3795.02	0.44	344	378	K.GMVLDENYNNLANAVISVSGINHDVTSGDHGDYFR.L
3811.01	3811.02	-0.01	344	378	K.GMVLDENYNNLANAVISVSGINHDVTSGDHGDYFR.L + Oxidation (M)
982.25	982.14	0.12	418	426	R.SIPQVSPVR.R

Table S17. Tryptic peptides from Spot #370 measured by MALDI-TOF. The protein was identified as complement C3C, N-terminal fragment, NCBI accession gi:78101270. Measured masses from the MALDI-TOF spectrum are compared to calculated values. Masses listed represent 4.8% sequence coverage. MS-Fit MOWSE score: 8.6e+03.

Measured mass, amu (average)	Calculated mass, amu (average)	error, amu	Sequence Position Start	End	Sequence
1816.76	1816.92	-0.15	1	16	-.SNLDEDIIAEENIVSR.S
2487.11	2486.86	0.25	66	86	K.GICVADPFEVTVMQDFFIDLR.L
2502.94	2502.86	0.08	66	86	K.GICVADPFEVTVMQDFFIDLR.L + Oxidation (M)
2070.39	2070.41	-0.02	114	131	K.VRVELLHNPAFCSLATTK.R
1304.61	1304.50	0.11	133	143	R.RHQQTVTIPPK.S
1471.70	1471.62	0.08	166	178	K.AAVYHHFISDGVR.K

Table S18. Tryptic peptides from Spot #458 measured by MALDI-TOF. The protein was identified as apolipoprotein A-I, SwissProt accession P02647. Measured masses from the MALDI-TOF spectrum are compared to calculated values. Masses listed represent 23% sequence coverage. MS-Fit MOWSE score: 9.8e+03; Mascot score: 50, expect value: 0.2.

Measured mass, amu (average)	Calculated mass, amu (average)	error, amu	Sequence Position Start	End	Sequence
1235.21	1235.43	-0.21	37	47	K.DLATVYYVDVLK.D
1400.22	1400.49	-0.27	52	64	R.DYVSQFEGSALGK.Q
1612.65	1612.73	-0.08	70	83	K.LLDNWDSVTSTFSK.L
1933.15	1933.08	0.07	86	101	R.EQLGPVTQEFWDNLEK.E
1252.33	1252.37	-0.04	121	130	K.VQPYLDDFQK.K

Table S19. Tryptic peptides from Spot #490 measured by MALDI-TOF. The protein was identified as apolipoprotein A-I, SwissProt accession P02647. Measured masses from the MALDI-TOF spectrum are compared to calculated values. Masses listed represent 23% sequence coverage. MS-Fit MOWSE score: 9.8e+03; Mascot score: 54, expect value: 0.09.

Measured mass, amu (average)	Calculated mass, amu (average)	error, amu	Sequence Position Start	End	Sequence
1235.14	1235.43	-0.28	37	47	K.DLATVYYVDVLK.D
1400.36	1400.49	-0.13	52	64	R.DYVSQFEGSALGK.Q
1612.67	1612.73	-0.06	70	83	K.LLDNWDSVTSTFSK.L
1933.01	1933.08	-0.07	86	101	R.EQLGPVTQEFWDNLEK.E
1252.13	1252.37	-0.24	121	130	K.VQPYLDDFQK.K
